# Prenatal acoustic communication triggers adaptive vascular programming in the developing avian brain

**DOI:** 10.1242/jeb.252287

**Published:** 2026-06-15

**Authors:** Prakrit Subba, Mylene M. Mariette, Katerina A. Palios, Michael G. Emmerson, Elisabetta Versace, Katherine L. Buchanan, David F. Clayton, Julia M. George

**Affiliations:** ^1^ Department of Biological Sciences, Clemson University, Clemson, SC 29634, USA; ^2^ Doñana Biological Station, EBD-CSIC, Seville 41092, Spain; ^3^ School of Life and Environmental Sciences, Deakin University, Waurn Ponds, VIC 3288, Australia; ^4^ School of Biological and Behavioral Sciences, Queen Mary University of London, London E1 4NS, UK; ^5^ Department of Genetics and Biochemistry, Clemson University, Clemson, SC 29634, USA

**Keywords:** Thermal physiology, Acoustic communication, Developmental plasticity, Developmental programming, Blood–brain barrier, Zebra finch

## Abstract

Developmental plasticity allows organisms to adjust their phenotypes to match environmental conditions, but how sensory cues program specific physiological systems remains poorly understood. In Australian zebra finches, incubating parents emit heat calls during extreme temperatures, and embryos exposed to these acoustic signals develop enhanced thermal tolerance and altered growth trajectories as adults, a striking example of anticipatory programming. We hypothesized that heat call exposure alters embryonic hypothalamic gene expression, given this brain region's central role in integrating environmental signals and regulating metabolism, thermoregulation and growth. We exposed zebra finch embryos to playback of parental heat calls or control calls during late incubation and used RNA-sequencing of hypothalamic tissue to identify transcriptional responses. Contrary to predictions of widespread neuroendocrine reprogramming, heat call exposure produced targeted changes: robust downregulation of genes regulating vascular smooth muscle contraction and cytoskeletal dynamics, with coordinated isoform switching. Cell-type analyses revealed these molecular changes localized to vascular endothelial cells, smooth muscle cells and ependymal cells, the cellular components that control cerebral blood flow and regulate the brain's vascular barrier. Gene expression patterns suggest increased vascular plasticity that may protect against heat-induced cellular damage. Remarkably, these adaptive modifications occurred in response to an acoustic signal alone, without thermal exposure. Our results provide transcriptional evidence that prenatal acoustic cues may program cerebrovascular function through cell type-specific gene regulation, providing a novel mechanism for sensory-mediated developmental plasticity. This targeted vascular programming may represent a conserved strategy for anticipatory adaptation to predictable thermal challenges across endothermic vertebrates.

## Teaser

**Highlighted Article:** Acoustic heat signals trigger transcriptional changes consistent with programming of brain vascular systems in zebra finch embryos, revealing how sensory cues trigger anticipatory adaptations to prepare organisms for environmental challenges.

## INTRODUCTION

How do animals prepare their offspring for the environmental challenges they will face after birth? Across taxa, prenatal environmental signals can program offspring phenotypes to match predicted conditions, triggering changes in developmental trajectories and resulting in long-term phenotypes, a process called developmental programming (Barker, 1990; Gluckman et al., 2005; Mariette et al., 2021; Monaghan, 2008; Nettle and Bateson, 2015). While biochemical cues such as maternal nutrition and hormones are well-established drivers of developmental programming (Bernardo, 1996; Groothuis and Schwabl, 2008; Mariette et al., 2021; Monaghan, 2008; Mousseau and Fox, 1998), the physiological mechanisms underlying other forms of environmental communication, particularly acoustic signals, remain largely unresolved (Mariette, 2024).

The Australian zebra finch (*Taeniopygia guttata*) provides a powerful model system for studying sensory-mediated developmental programming. Adult zebra finches produce distinctive heat calls when exposed to high ambient temperatures, with increased calling rates during late incubation when embryos may perceive sound (Mariette and Buchanan, 2016, 2019; McDiarmid et al., 2018). These parental vocalizations appear to forecast thermal conditions that offspring will experience upon hatching (Mariette, 2020). Late-stage embryos exposed to heat calls prenatally display adaptive phenotypic changes postnatally, including altered growth rates, begging call production and mitochondrial function as nestlings, as well as modified thermal preferences, enhanced heat tolerance and increased reproductive success as adults (Mariette and Buchanan, 2016; Pessato et al., 2022; Udino and Mariette, 2022; Udino et al., 2021). Thus, prenatal acoustic cues can produce developmental changes in behavior, morphology and physiology that appear adaptive for predicted environmental conditions (Mariette et al., 2021). However, the physiological mechanisms through which acoustic programming operates remain unknown.

We hypothesized that the postnatal phenotypic changes induced by prenatal heat call exposure would be preceded by changes in hypothalamic gene expression upon receiving the acoustic cue. The hypothalamus is a central integrator of environmental signals and neuroendocrine regulators of metabolism, thermoregulation and growth (Debonne et al., 2008; Groothuis et al., 2019; Sheng et al., 2020). Across vertebrates, early-life environmental cues can reprogram the hypothalamic–pituitary–adrenal (HPA) axis, with lasting consequences for glucocorticoid physiology and downstream developmental trajectories (Plotsky and Meaney, 1993; Welberg and Seckl, 2001). In birds, maternal hormones and prenatal sensory signals can modulate developmental outcomes via the HPA axis (Kraft et al., 2021; Ruiz-Raya et al., 2023). For instance, yellow-legged gull (*Larus michahellis*) embryos perceive prenatal cues such as adult alarm calls and shifts in light exposure and respond with coordinated changes in corticosterone levels and glucocorticoid receptor expression (Noguera and Velando, 2019; Ruiz-Raya et al., 2023). However, evidence directly linking such HPA axis modifications to changes in hypothalamic gene regulation remains limited (Avishai-Eliner et al., 2001; Korosi et al., 2010; Marasco et al., 2016; Yan et al., 2020; Zimmer and Spencer, 2014). We therefore targeted the hypothalamus to test our primary hypothesis that neuroendocrine pathways mediate the thermoregulatory programming of traits observed in nestlings and adults.

Beyond systemic neuroendocrine programming, we considered a complementary hypothesis: upon receiving an acoustic signal of impending heat stress, the brain itself, one of the most heat-sensitive organs in endotherms (Bain et al., 2015; Yoneda et al., 2024), may undergo local physiological adjustments to withstand elevated temperatures. Under hyperthermia, the mechanisms enabling brain cooling or preventing cellular damage are not fully elucidated in birds and mammals but involve modulation of cerebral blood flow and oxygen delivery (Bain et al., 2015; Porter and Witmer, 2016). Across taxa, cerebral blood flow is regulated by diverse vascular-associated cells, including endothelial cells that line blood vessels and form the brain's vascular barrier, contractile mural cells (vascular smooth muscle cells and pericytes) and glial cells (astrocytes and microglia) (Bain et al., 2015; McConnell and Mishra, 2022; Paton et al., 2023). These cells exhibit remarkable phenotypic plasticity in response to environmental stressors, including thermal challenges (Hayes et al., 2022; Phan et al., 2025). In mammals, severe heat stress causes vascular damage, hemorrhage and inflammation in the hypothalamus (Yoneda et al., 2024). Importantly, cerebrovascular systems are also subject to developmental programming (Smolders et al., 2018), with recent evidence confirming that early-life stress can have lifelong effects on barrier function by altering its developmental trajectory (Zhao et al., 2022). Therefore, we tested the non-mutually exclusive hypothesis that heat calls trigger anticipatory modifications to cerebrovascular cells, preparing the hypothalamus to withstand future thermal stress.

To test whether embryonic exposure to heat calls influences hypothalamic gene expression, we exposed zebra finch eggs to playback of parental heat calls or control calls during late-stage incubation, then isolated hypothalamic tissue and performed RNA-sequencing. To our knowledge, this study provides the first comprehensive transcriptomic analysis of the embryonic songbird hypothalamus in response to any prenatal environmental signal. To identify coordinated changes in gene expression that would reveal the endocrine or other functional pathways underlying developmental programming and postnatal phenotypes, we conducted genome-wide differential expression analysis and weighted gene co-expression network analysis (WGCNA). We complemented these approaches with a targeted analysis of genes with established hypothalamic neuroendocrine functions to test our primary hypothesis. To test our secondary hypothesis of localized cerebrovascular adaptations, we used the genome-wide analysis to systematically interrogate gene expression changes between playback groups, focusing on both individual genes and isoform-level shifts, followed by cell-type enrichment analysis to identify which hypothalamic cell populations showed transcriptional responses. Our integrative approach provides insights into how prenatal acoustic cues program physiological systems and reveals novel cellular targets for anticipatory thermal adaptation.

## MATERIALS AND METHODS

### Experimental design

To obtain eggs for playback treatment, adult zebra finches, *Taeniopygia guttata* (Vieillot 1817), were allowed to pair and breed in an outdoor aviary at Deakin University. Eggs collected on the laying day were placed in a fanless artificial incubator (Bellsouth 100 electronic incubator) at 37.5°C for 9 days at 60% humidity. On day 10 (E10) in the morning, the eggs were randomly allocated, using a within-clutch design and balancing laying order, to one of two experimental incubators, each equipped with a different acoustic playback treatment: control call playback (whine calls combined with tet calls, *n*=9) or heat call playback (whine calls combined with heat calls, *n*=10). We included 9–10 biological replicates per treatment group, which is comparable to or exceeds sample sizes commonly reported in adult zebra finch hypothalamus studies (*n*=4–6 per group) (Kumari et al., 2022). Whine calls are contact calls with a complex acoustic structure that may contribute to auditory system development, while tet calls are typical contact calls commonly uttered by parents at the nest but not related to temperature (Boucaud et al., 2016). By contrast, heat calls are fast, high-pitched vocalizations produced by adult zebra finches under thermal stress (Pessato et al., 2020). Prenatal acoustic playbacks were broadcast daily from E10 to E13 for 9 h per day (10:00–19:00 h) at 65 dB sound pressure level (SPL). The playback volume (65 dB SPL) was selected to approximate the sound level of typical zebra finch calls (including control contact calls) (Elie and Theunissen, 2016). Heat calls are likely softer, although their sound level has not been measured accurately and appears to vary between contexts, being louder during incubation (M.M.M., personal observation). Importantly, under natural incubation conditions, eggs are in direct physical contact with the calling adult, with sound traveling directly from ‘solid’ to ‘solid’ (i.e. body tissues), without any barrier (including air) to sound transmission; and avian embryos are understood to perceive sound through bone conduction (Rumpf and Tzschentke, 2010). Sound levels measured 10 cm from the emitter (i.e. after crossing the boundary from solid to air) would therefore greatly underestimate the level experienced by naturally incubated embryos. Playback temporal structure mimicked that of naturally vocalizing adults in the nest, with playback restricted to the active and warmer phase (10:00–19:00 h) and call sequences interspaced with periods of silence, consistent with the diurnal pattern of heat call and control call production observed in wild zebra finches (Mariette et al., 2018). Playbacks were delivered via speakers (Sennheiser HD439, Video Guys, Melbourne, VIC, Australia) positioned inside each experimental incubator and externally connected to an amplifier (Digitech 18 W, Jaycar, Geelong, VIC, Australia) and audio player (Zoom H4nSP and Marantz PMD670, Video Guys). To control for potential incubator-specific effects, egg trays and audio playback equipment were swapped daily between the two experimental incubators. On the night of E13, embryos from both playback treatments were transferred to a single shared silent incubator to equalize the immediate pre-sampling environment.

On the morning of E14 (i.e. day before hatch) between 10:00 h and 12:00 h, embryos were extracted from eggs and euthanized by rapid decapitation. Isolated heads were immediately embedded in optimal cutting temperature (OCT) compound and flash-frozen on dry ice. Frozen tissue blocks were shipped on dry ice from Deakin University to Queen Mary University of London and stored at −80°C upon arrival until cryosectioning for hypothalamic tissue collection. Brain tissue was sectioned coronally at 100 μm thickness on a cryostat, and a single cylindrical punch (1 mm diameter) was collected when the third ventricle became visible, approximately 5000 μm caudal to the beak. Prior to sampling experimental embryos, we validated that punches captured hypothalamic tissue by performing fluorescence *in situ* hybridization with hypothalamic gene markers in whole-brain coronal sections of untreated embryos (Fig. S3). Tissue punches were immediately collected into cryotubes on dry ice and stored at −80°C until RNA extraction. All procedures were approved by the Animal Ethics Committee of Deakin University (G29-2016) and conducted in accordance with Australian guidelines for animal research.

### RNA extraction, sequencing and statistical analysis

Total RNA was extracted using the Zymo Quick-RNA Microprep Kit (cat. no. R1050) at the Genome Centre, Queen Mary University of London. RNA quality and concentration were assessed using NanoDrop spectrophotometry and an Agilent 2100 Bioanalyzer. The RNA-Seq experiment was conducted on the Illumina NextSeq 500 platform in 75 base pair, paired-end mode. Quality control was performed by trimming low quality sequences using Trimmomatic (v0.39) (Bolger et al., 2014). The RNA-Seq reads were quantified with Salmon v1.5.2 using a decoy-aware gentrome index built from the zebra finch reference transcriptome (bTaeGut1.4.pri) concatenated with the genome. Salmon was run in selective-alignment mode (–validateMappings) on paired-end reads with automatic library-type detection (–libType A) and 8 threads; otherwise, default parameters were used. No additional uniquely mapped-read filter was applied, as Salmon incorporates multimapping reads during transcript quantification through its probabilistic assignment framework (Patro et al., 2017). A total of 49,298 transcripts corresponding to 21,407 genes were detected and quantified across all samples. Gene-level counts were normalized and modeled using a negative binomial generalized linear model in the R package DESeq2 (v1.30.1) (Love et al., 2014). Genes with fewer than 10 counts summed across all 19 samples were excluded prior to modeling. All 19 embryos passed quality control and were included; no samples were excluded *post hoc*. Sex was determined directly from the RNA-Seq data by examining the expression of sex chromosome-linked marker genes: Z-linked genes (*HINT1* and *CHD1*), which show lower abundance in females (ZW) relative to males (ZZ), and W-linked genes (*CHD1W* and *LOC100190380*), which are absent in males. This approach is standard in avian embryonic transcriptomics where morphological sex determination is unreliable. The primary model tested the main effect of treatment (∼ sex+playback condition) using a likelihood ratio test (LRT) against a reduced model containing only sex. Log_2_-fold changes were shrunk using apeglm to stabilize variance estimates and improve ranking. Significant differentially expressed genes (DEGs) were defined as those with a Benjamini–Hochberg adjusted *P*-value (*P*_adj_)<0.05. The final set comprised 49 DEGs (48 downregulated, 1 upregulated). Secondary analysis assessed the interaction term (∼ sex+playback condition+sex:playback condition) to investigate the sex-by-treatment interaction at *P*_adj_<0.05. Principal component analysis (PCA) was performed on variance-stabilization transformed (VST) data to characterize total expression variance.

The differential expression analyses in DESeq2 used negative binomial generalized linear models with gene-wise dispersion estimates and mean-variance trends fitted from the data. All *P*-values obtained from the Wald and likelihood ratio tests were adjusted for multiple comparisons using the Benjamini–Hochberg false discovery rate (FDR) method. VST was applied to normalized counts to obtain approximately homoscedastic, log-like expression values for PCA and downstream correlation-based analyses (see ‘Weighted gene co-expression network analysis’, below).

To complement the genome-wide approach, we performed targeted differential expression analysis on a literature-derived set of 143 genes with established hypothalamic function (Table S1). This gene list was compiled from published expression atlases and functional studies of the avian and mammalian hypothalamus (Antin et al., 2007; Bell et al., 2004; Lovell et al., 2020; Rouillard et al., 2016; Uhlén et al., 2015). The full DESeq2 dataset (19,137 genes) was subset to include only those hypothalamic genes present in the filtered count matrix. Differential expression was then tested on this reduced gene set using DESeq2 with the same model specification (∼ sex+playback condition) and likelihood ratio test framework as the genome-wide analysis. Log_2_-fold changes were estimated using apeglm shrinkage. Multiple testing correction was applied via the Benjamini–Hochberg method within the hypothalamic gene subset. This targeted approach reduces the multiple-testing burden relative to the whole-transcriptome analysis, thereby increasing sensitivity to detect modest expression changes in genes of *a priori* biological interest.

To identify biological pathways and functional categories associated with heat call exposure, DEGs were subjected to Gene Ontology (GO) and KEGG pathway enrichment analysis for biological processes using ShinyGO v0.85.1 (species: *Homo sapiens*) (Ge et al., 2020). To mitigate incomplete functional annotation in the zebra finch genome, we mapped zebra finch genes to human homologs using the biomaRt R package (v2.60.1) to query the *Taeniopygia guttata* Ensembl dataset (tguttata_gene_ensembl; release 110) (Kinsella et al., 2011). For each gene, we retrieved the precomputed human homolog field (hsapiens_homolog_associated_gene_name) from Ensembl Compara via BioMart, where orthologs are inferred from gene trees and supported by additional comparative evidence including gene order conservation and whole-genome alignment coverage (Herrero et al., 2016). Genes without an annotated human homolog were excluded, and duplicate human gene symbols were collapsed to unique identifiers prior to enrichment testing. Zebra finch genes with identifiable human orthologs were used as input for functional enrichment analysis. To ensure the statistical universe matched the set of genes measurable in our experiment, we provided a custom background gene set consisting of all 21,407 zebra finch genes included in the RNA-Seq analysis that successfully mapped to human orthologs (*n*=11,310 unique human genes). Enrichment was tested against GO Biological Process, GO Molecular Function, GO Cellular Component and KEGG pathway databases. Enrichment *P*-values were calculated using the hypergeometric test and corrected for multiple testing using the Benjamini–Hochberg FDR method. Pathways meeting FDR<0.05 were considered statistically significant. Pathway size limits were set to a minimum of 2 and a maximum of 5000 genes. Redundancy reduction was enabled to collapse highly overlapping pathways (95% gene overlap and 50% name overlap). Results were ranked by fold-enrichment within the FDR-significant set, and the top pathways per module were reported as bar plots displaying fold-enrichment and FDR values.

### Isoform switch analysis

To identify playback condition-wise splice-isoform usage patterns in our RNA-Seq data, we used the IsoformSwitchAnalyzeR package in R (v2.4.0) to perform an isoform switching analysis (Vitting-Seerup and Sandelin, 2019). A design matrix with sound playback conditions and sex as covariates was constructed from sample metadata (*n*=19). We identified transcripts at the isoform resolution by using a gene transfer format (GTF) and transcript FASTA files (bTaeGut1.4.pri assembly). We filtered the transcript isoforms of genes by removing genes with (i) expression <1 transcript per million (TPM), (ii) isoforms with 0 TPM and (iii) single-isoform genes. Differential isoform usage was quantified using DEXSeq (α=0.05 FDR adjusted; differential isoform fraction ≥0.05) using the isoformSwitchTestDEXSeq function. We characterized splicing events using two complementary approaches: at the genome level (via extractSplicingGenomeWide) and at the event or mechanistic level (via extractSplicingEnrichment). We used the default junction overlap thresholds to calculate intron retention frequencies in transcripts using the analyzeAlternativeSplicing function. Finally, changes in isoform usage and expression between control and heat call playback conditions were visualized.

### Weighted gene co-expression network analysis

To identify patterns of gene co-expression and their relationships with different embryonic sound playbacks, we conducted a weighted gene co-expression network analysis (WGCNA) using the WGCNA (v1.73) package in R (Langfelder and Horvath, 2008). The input transcript read counts were prepared based on the zebra finch reference transcriptome (GCF_003957565.2_bTaeGut1.4.pri assembly) using Salmon v1.5.2 (Patro et al., 2017). These gene expression raw counts were normalized using the VST method in the DESeq2 package. A DESeq2 dataset was constructed with intercept only in the design formula. The VST normalization was applied ignoring the experimental design to produce variance-stabilized, log-like expression values. The resulting normalized expression matrix was transposed in a sample-by-genes format for downstream analyses. Networking construction parameters such as the soft-thresholding power were selected using an iterative approach. The scale-free topology fit index (*R*^2^) and mean connectivity were calculated for a range of powers from 1 to 30 to find the optimal power (power=8) achieving a high scale-free topology fit. The network was constructed using a minimum module size of 30 genes and a module similarity (i.e. merge cut height) of 0.25.

To examine module–trait relationships, module assignments were converted to color labels, module eigengenes were calculated, and Pearson correlations were computed between eigengenes and sex or playback condition using VST-normalized expression values. Significance of these correlations was assessed using two-sided Student's t-tests, and results were visualized as a heatmap annotated with correlation coefficients and corresponding *P*-values. We performed a GO and KEGG pathway enrichment analysis as described previously (see ‘RNA extraction, sequencing and statistical analysis’, above) for modules significantly correlated with heat call playback. For each module, genes with identifiable human orthologs were used as input for functional enrichment analysis. Results were reported as bar plots displaying fold-enrichment and FDR values.

To find the hub genes in each module, we calculated the intramodular connectivity (kWithin) for each gene using the adjacency matrix raised to the selected-threshold power. For the green module, edges with adjacency weights below 0.4 were excluded to focus on robust co-expression patterns. The top five hub genes, with the highest kWithin calculated from the adjacency matrix, were identified for the green module. These hub genes and their associated neighbors were visualized as network layouts using the Kamada–Kawai algorithm using the ggraph package (v2.2.1) (https://github.com/thomasp85/ggraph/releases/tag/v2.2.1).

### Cell-type enrichment analysis

To establish cellular specificity of the identified modules from the WGCNA analysis, we conducted a cell-type enrichment analysis using a publicly available dataset ‘HYPOMAP: A comprehensive spatio-cellularmap of the human hypothalamus’ available at the CZ CELLxGENE Discover database (CZI Cell Science Program, 2025; Tadross et al., 2025). A MAST test was conducted using the FindAllMarkers function in the Seurat R package (v5.2.1) (Hao et al., 2024) to identify the top 100 cell-type marker genes with minimum expression in 25% of cells and a minimum log fold-change threshold of 0.25. These marker genes were converted from ensembl identifiers to human gene symbols using the org.Hs.eg.db annotations (https://bioconductor.org/packages/org.Hs.eg.db/). Next, the zebra finch genes co-expressed in the WGCNA green module were mapped to their human orthologs using the biomaRt R package (v2.60.1) interfaced with Ensembl (release 110, accessed July 2025) (Kinsella et al., 2011). Finally, cell type-module associations were quantified using a hypergeometric test by comparing each module's gene set against cell type markers. The *P*-values were adjusted for multiple-testing correction using the Benjamini–Hochberg method and the results were visualized using a heatmap.

### Deconvolution analysis

To estimate the cellular composition of our bulk-tissue RNA-Seq data and obtain cell type-specific abundance, we used CIBERSORTx (v1.0) (Newman et al., 2019). Bulk tissue mixture files containing transcript-level normalized counts (counts per million, CPM) were prepared according to the package's recommendations. A custom signature matrix was generated from a single-cell RNA-sequencing atlas of the human hypothalamus (HYPOMAP; CZI Cell Science Program, 2025; Tadross et al., 2025). The cell fractions mode was used to construct a batch-corrected signature matrix and adjusted bulk mixture files with 500 permutations and without quantile normalization using the Docker version of CIBERSORTx. These batch-corrected files were subsequently uploaded to the CIBERSORTx web portal to run a custom cell fractions analysis with 1000 permutations in absolute mode, and the output files were visualized using R.

CIBERSORTx was run in absolute mode, which reports RNA-based abundance scores for each cell type that scale with an overall ‘absolute score’ per sample and are therefore not constrained to sum to 1. To derive within-sample cell-type proportions, absolute-mode scores were normalized by dividing each cell-type value by the sample's absolute score, such that relative fractions sum to 1 across the cell types represented in the signature matrix. For each sample, CIBERSORTx reported a goodness-of-fit *P*-value, the Pearson correlation between observed and model-predicted bulk expression, and the root mean square error (RMSE). In this dataset, all samples had *P*<0.0001, Pearson *r*>0.89, and RMSE between 0.70 and 0.75 (Table S6).

### Methods for hybridization chain reaction

To validate the presence of hypothalamic tissue in the regions surrounding the third ventricle, we performed hybridization chain reaction (HCR) for the *SIM1* gene (single-minded bHLH transcription factor 1), a marker for the paraventricular nucleus (PVN) identified from single-nucleus RNA sequencing data of zebra finch hypothalamus (P.S., Vijay Shankar, Kaitlyn Williams, Trudy F. C. Mackay, Patrick S. Freymuth, Natalie A. Shay, Allison M. Rees, Shannon R. Liedl, Jonathan Roberts, D.F.C and J.M.G, unpublished results). Coronal sections of E13 embryos from three untreated animals were collected caudal to the eye, where the third ventricle becomes visible. Brains were sectioned at 20 μm using a Leica CM3050 S cryostat (Leica Microsystems, Wetzlar, Germany) and mounted on Superfrost Plus slides. Tissue sections were immediately fixed in 4% paraformaldehyde, hydrated in 1× PBS, dehydrated through a 70%, 95% and 100% ethanol series, and stored at −20°C until the following day for hybridization.

Fixed tissue sections were processed for HCR™ Gold RNA-FISH following the manufacturer's sample-on-slide protocol, with minor modifications (Choi et al., 2010, 2014, 2016, 2018). Notably, samples were thawed and rehydrated before probe hybridization; the protocol was optimized by extending probe incubation to overnight and amplification to 3 h. Sections were then co-stained with DAPI (1 μg ml^−1^) and imaged using the Leica SR GSD widefield super-resolution microscope with TIRF module (Leica Microsystems).

Fluorescence images were acquired as single-plane TIFF files using Leica LAS X software on the Leica SR GSD widefield microscope (no Z-stacks or deconvolution were applied). Raw TIFF images were imported into FIJI/ImageJ (v2.16.0/1.54p) and converted to 16-bit grayscale prior to visualization (Schindelin et al., 2012). For display, individual channels (*SIM1* probe signal and DAPI) were pseudocolored (*SIM1*: magenta; DAPI: cyan) and merged to generate composite images. Only linear adjustments to brightness and contrast were applied uniformly across each image, and no further image processing, filtering or quantitative analysis was performed.

### Use of AI tools

Large language models accessed via the Perplexity platform (including Grok 4.1, GPT 5.1, Sonar and Claude Sonnet 4.5) were employed to assist with R script optimization and debugging of computational workflows, including RNA-Seq, WGCNA, isoform switching and deconvolution pipelines. These models were also used to help edit and refine portions of the manuscript, particularly the Materials and Methods and figure legends, to ensure adherence to the target journal's formatting and reporting guidelines. All AI-assisted code and text were critically reviewed, modified and validated prior to inclusion in the manuscript, and the authors take full responsibility for the accuracy and integrity of all content. No AI tools were used for image creation, editing or enhancement.

## RESULTS

### Targeted analysis of hypothalamic neuroendocrine genes

**Fig. 1.**
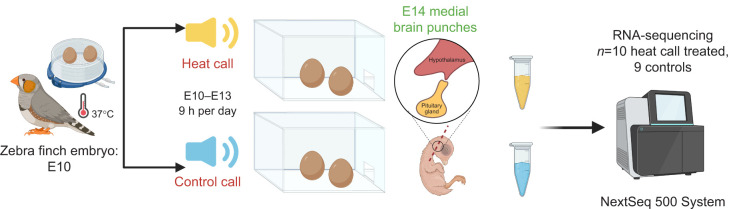
**Experimental design for RNA-sequencing in late-stage zebra finch embryos.** Zebra finch embryos were exposed to chronic heat call (*n*=10) or control call (*n*=9) playback from incubation day 10 (E10) to E13. Hypothalamic tissue collection occurred on E14, followed by RNA-sequencing and analysis. Created using BioRender.com by Subba, P. (2026). https://BioRender.com/k5anlxi. This figure was sublicensed under CC-BY 4.0 terms.

Medial hypothalamic brain punches were collected from E14 embryos (i.e. 1 day before hatch) that had been exposed to playbacks of either heat calls or control calls for 9 h per day from incubation day 10 (E10) to 13 (E13) (*n*=10 heat call- and 9 control call-exposed embryos; Fig. 1). A total of 19 embryos were included: 10 exposed to heat call playback (*n*=7 female, *n*=3 male) and 9 controls (*n*=2 female, *n*=7 male). To test our primary hypothesis that heat call exposure modulates neuroendocrine gene regulation, we compiled a literature-derived list of 143 hypothalamic genes with established roles in neuroendocrine function (Table S1) and performed a targeted differential expression analysis on this subset. This focused approach reduces the multiple-testing penalty inherent in whole-transcriptome analyses while maintaining statistical rigor. No genes reached significance at an FDR threshold of 0.05. Nonetheless, 12 genes showed differential expression at an unadjusted significance threshold (Wald test *P*<0.05), although adjusted *P*-values exceeded 0.25 (Fig. S1). Among these candidates, the glucocorticoid receptor gene *NR3C1* showed the strongest evidence for modulation, with reduced expression in heat call-exposed embryos relative to control call playbacks [shrunken log_2_-fold change (LFC)=−0.26, *P*=0.0045, FDR=0.25]. Aryl hydrocarbon receptor nuclear translocator gene *ARNT* was also downregulated (shrunken LFC=−0.13, *P*=0.0088, FDR=0.25). In contrast, several neuroendocrine genes showed modest increases in expression following heat call exposure: corticotropin-releasing hormone receptor 1 gene *CRHR1* (shrunken LFC=0.068, *P*=0.013, FDR=0.29), growth hormone-releasing hormone gene *GHRH* (shrunken LFC=0.021, *P*=0.0083, FDR=0.25), neurotensin gene *NTS* (shrunken LFC=0.051, *P*=0.0075, FDR=0.25) and somatostatin gene *SST* (shrunken LFC=0.066, *P*=0.027, FDR=0.42) (Fig. S1 and Table S1). These changes did not reach significance after correction for multiple testing and thus do not statistically support widespread neuroendocrine reprogramming of the hypothalamus at this embryonic stage.

### Heat call exposure downregulates vascular smooth muscle contractile programs

Across the 19 samples, RNA-Seq yielded a mean effective library size of 33.8 million gene-level counts (median 31.8 million; range 26.4–62.6 million; *n*=19 individuals), and 19,137 genes with at least 10 total counts were retained for differential expression analysis. To characterize global gene expression patterns in the dataset, PCA of variance-stabilized counts (19,137 genes, 19 samples) revealed pronounced transcriptomic sex differentiation, with PC1 (48% of variance) strongly separating males and females (*r*=0.989; Fig. S2). To our knowledge, this represents the first demonstration of sex-differentiated gene expression in the embryonic songbird hypothalamus. Beyond sexual differentiation, both PC1 and PC2 (17% of variance) captured moderate separation by playback condition (*r*=−0.549 and *r*=0.477, respectively), with only weak association with sex for PC2 (*r*=0.117; Fig. S2). Therefore, beyond sex differences, prenatal acoustic experience explained a noticeable amount of variation in overall hypothalamic gene expression.

**Fig. 2.**
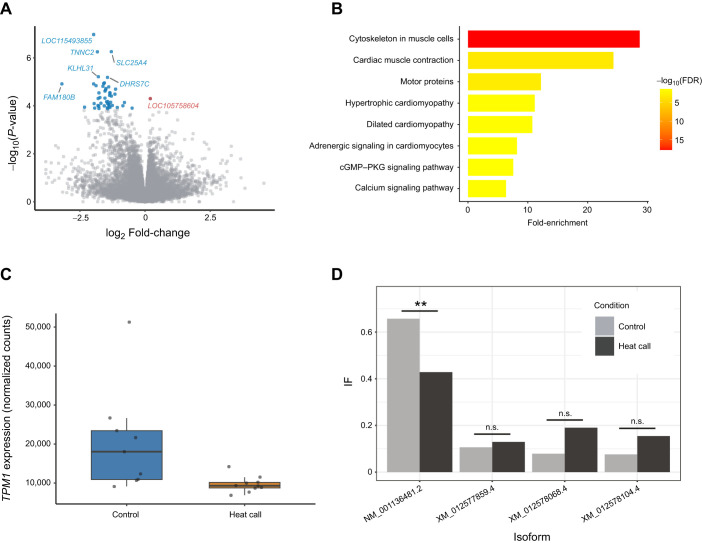
**RNA-Seq analysis of medial hypothalamic punches from late-stage zebra finch embryos exposed to chronic heat call or control call playback.** Medial hypothalamic punches were collected from *n*=10 heat call and *n*=9 control embryos (one punch per embryo; 19 biological replicates). (A) Volcano plot of treatment effect on gene expression [log_2_-fold change (*x*-axis) and −log_10_(*P*-value) (*y*-axis)] for individual genes; blue and red points indicate genes with Benjamini–Hochberg adjusted *P*-value (*P*_adj_)<0.05 from DESeq2 likelihood ratio tests (blue, downregulated; red, upregulated; 49 DEGs total: 48 downregulated, 1 upregulated). (B) Pathway enrichment of treatment-responsive differentially expressed genes (DEGs) analyzed in ShinyGO (v0.85.1, KEGG database) using hypergeometric tests with Benjamini–Hochberg false discovery rate (FDR) correction; bars show fold-enrichment, color encodes −log_10_(FDR). (C,D) Nominal downregulation of *TPM1* expression (DESeq2 *P*_adj_=0.064; C) and differential isoform usage (D), shown as isoform fraction (IF; *y*-axis) for individual *TPM1* transcripts (*x*-axis) in control (gray) and heat call (black) conditions, with significant differential isoform usage from IsoformSwitchAnalyzeR DEXSeq tests (two-sided FDR corrected; **q<0.01, |ΔIF|≥0.05).

Using a DESeq2 model including sex and playback condition (∼ sex+playback condition), 49 genes were differentially expressed between heat call and control embryos (*P*_adj_<0.05). The response was strongly asymmetric, with 48 genes downregulated and 1 gene upregulated in heat call-exposed embryos relative to control call playback (Fig. 2A; Table S2). A secondary model including the interaction term (∼sex+playback condition+sex:playback condition) identified five genes with significant sex-by-treatment interactions (*P*_adj_<0.05): three sex-linked genes (*HDHD2* on the Z chromosome; *LOC116806857* and *LOC116806879*/*KCMF1* on the W chromosome) and two autosomal genes (*ABRACL* and *PTPRM*), indicating that some transcriptional responses to heat call exposure differ between males and females.

Contrary to our predictions of neuroendocrine reprogramming, heat call exposure primarily affected genes regulating vascular smooth muscle contraction and cytoskeletal organization. Pathway enrichment analysis of the 48 downregulated genes revealed significant over-representation of contractile and cytoskeletal programs (enrichment FDR<0.05; Fig. 2B). The top enriched terms were Cytoskeleton in muscle cells (FDR<0.01; fold-enrichment=28.69), Cardiac muscle contraction (FDR<0.01; fold-enrichment=24.30) and Motor proteins (FDR<0.01; fold-enrichment=12.20). These pathway-level signals correspond to downregulation of multiple canonical contractile and cytoskeletal genes, including *TPM1*/*TPM2*/*TPM4*, *ACTC1*/*ACTA1*, *MYL2*/*MYL3*, *TNNC1*/*TNNT2*, *DES* and *LMNA* (Table S3). Together, these results indicate that a focused transcriptional program regulating vascular smooth muscle function is selectively repressed in the medial hypothalamus following prenatal heat-call exposure. Given that vascular smooth muscle cells (VSMCs) and pericytes collectively regulate cerebral blood flow and vascular tone (Paton et al., 2023), this response suggests targeted modulation of cerebrovascular regulation rather than widespread hypothalamic reprogramming.

### Heat call exposure alters isoform usage in contractile genes

Even with equivalent total gene expression, differences in which isoform is produced between treatment groups can substantially alter protein function and cellular phenotype (Graveley, 2001). To investigate transcript isoform changes associated with heat call exposure, we conducted an isoform switch analysis using the transcriptome-wide RNA-Seq data. After filtering to remove 20,682 low-expression and single-isoform gene transcripts, we investigated differential isoform usage in 23,054 of the remaining isoforms. A total of 26 genes showed significant treatment-specific isoform switching between playback treatments (FDR<0.05; differential isoform fraction ≥0.05) (Table S4). Overall, exon skipping was the dominant alternative splicing event, accounting for 17 events among the 26 switching isoforms (Table S5). Within these, most genes showed decreased exon skipping (i.e. increased exon inclusion) in heat call-treated embryos, with the enrichment analysis showing a nominally significant difference (proportion of gains=0.24, *P*=0.049, *q*=0.34; total exon skipping events=17) (Table S5). Other alternative splicing events (alternative splice sites, transcription start/stop sites, mutually exclusive exons, and intron retention) were less common and did not show significant differences between treatments (all *q*≥0.77) (Table S5).

Among the significant switches, three genes showed especially strong differential isoform usage between playback groups: *TLK2*, involved in DNA repair and chromatin maintenance (*q*<0.001, ΔIF=−0.06), *LOC100228270* (*q*=0.009, ΔIF=−0.26) and *TPM1*, encoding Tropomyosin 1 involved in cytoskeleton and muscle contraction, and whose excess has been linked to neuroinflammation by microglia activation (*q*=0.009, ΔIF=−0.23; Fig. 2D; Table S4) (Li et al., 2022a,b). All three genes displayed a lower fraction of the most common isoform in heat call-exposed embryos compared with control call playback. Notably, *TPM1* also showed borderline gene-level downregulation (log_2_ fold-change=−1.11, *P*_adj_=0.064, baseMean=14,769.90; Fig. 2C), providing convergent evidence for reduced *TPM1* activity through both overall expression and isoform usage following prenatal heat call exposure. This coordinated regulation suggests post-transcriptional fine-tuning of vascular contractile programs in response to acoustic developmental programming.

### Co-expression network analysis reveals coordinated vascular gene regulation

**Fig. 3.**
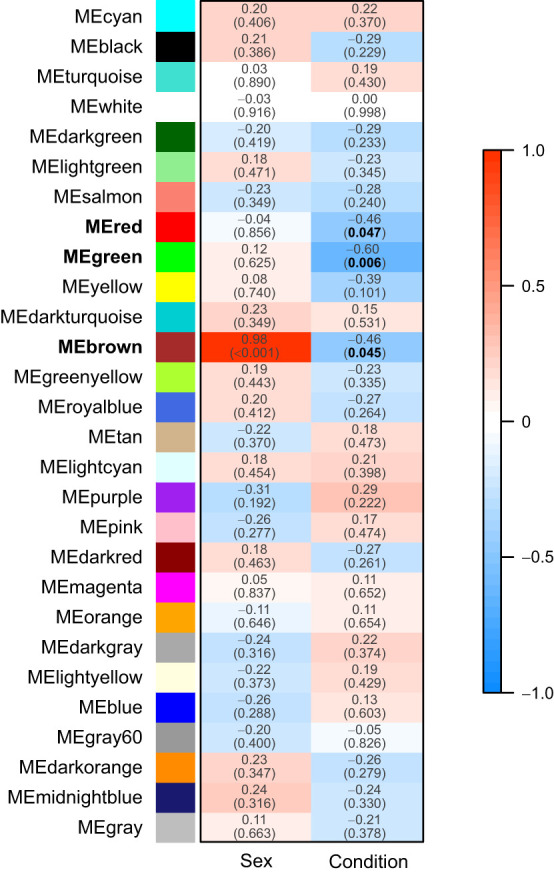
**Module–trait relationships between WGCNA eigengenes and sex or playback condition.** Heatmap showing Pearson correlation coefficients (cell values) and two-sided Student's *t*-test *P*-values (in parentheses; bold indicates significance) between WGCNA module eigengenes and sex (left) or playback condition (right), derived from medial hypothalamic RNA-Seq (*n*=19: 10 heat call, 9 control embryos), with males and control call used as the reference, respectively. Color intensity reflects association strength and direction (red, positive; blue, negative).

To assess possible functional changes, we identified coordinated changes in hypothalamic gene expression at the level of biological pathways by conducting a WGCNA. We used DESeq2 variance-stabilized expression values for 21,407 genes detected in the medial hypothalamus RNA-Seq dataset across 19 samples. All genes detected by the gene-level quantification step were retained for co-expression network construction, without additional variance-based pre-filtering, and module detection was performed on this full gene set. We identified 28 distinct modules, each containing a group of highly correlated genes with similar expression patterns across samples, with sizes ranging from 76 to 5629 genes per module. Modules were automatically assigned arbitrary color labels by WGCNA (e.g. green, red, brown), which serve as identifiers only and have no inherent biological meaning (Fig. 3). In our module–trait correlation analysis, the green module (*n*=463 genes) showed the strongest negative correlation with heat call response (*r*=−0.60, *P*<0.01) (Fig. 3). Additionally, the brown (*r*=−0.46, *P*=0.047) and red (*r*=−0.46, *P*=0.045) modules were negatively correlated with heat call playbacks (Fig. 3). The red module also contained genes related to muscle contraction and development (Table S3). The brown module (*n*=1363 genes) showed the strongest correlation with sex, indicating upregulation in females (*r*=0.98, *P*<0.001) (Fig. 3). No other modules had significant module–trait correlations.

**Fig. 4.**
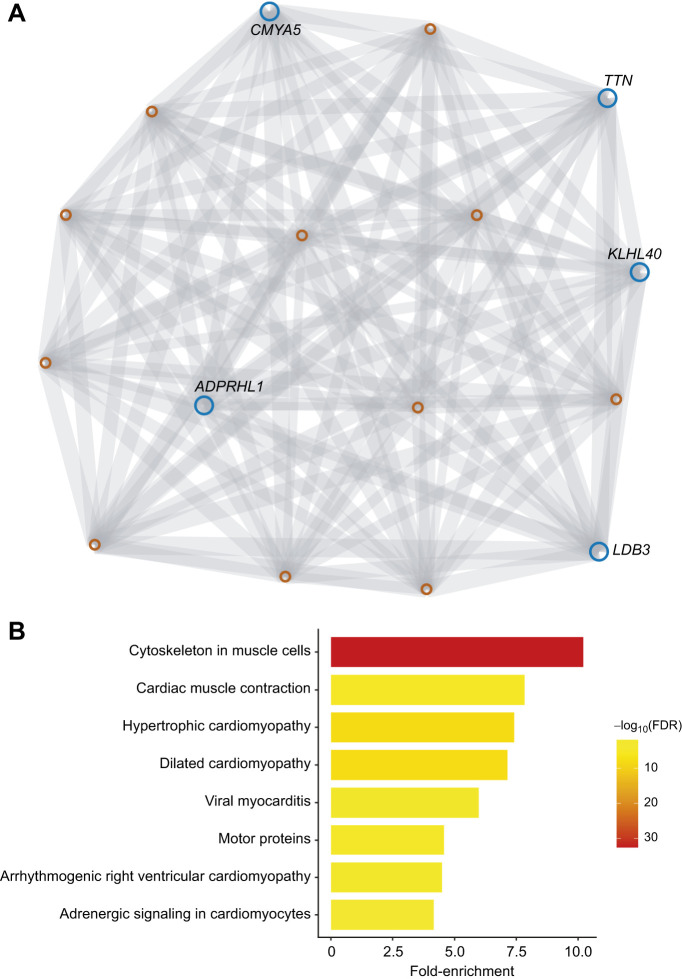
**Green WGCNA module structure and functional enrichment.** (A) Green module co-expression network from WGCNA of hypothalamic RNA-Seq data (*n*=19 embryos: 10 heat call, 9 control) showing the top five hub genes (larger blue circles; ranked by intramodular connectivity kWithin) and their strongest co-expressed neighbors (smaller red circles; adjacency>0.4). Edge thickness indicates pairwise co-expression strength. (B) Pathway enrichment analysis for green module genes analyzed in ShinyGO (v0.85.1, KEGG database) using hypergeometric tests with Benjamini–Hochberg FDR correction; bars show fold-enrichment, color encodes −log_10_(FDR).

GO term analysis revealed the biological functions associated with modules correlated with heat call playback. The green module was significantly enriched in terms including Myofibril assembly (GO:0030239), Cytoskeleton in muscle cells (Path:tgu04820), Muscle structure development (GO:0061061) and Contractile muscle fiber (GO:0043292) (FDR<0.01), indicating coordinated downregulation of genes involved in contractile and cytoskeletal programs (Fig. 4B). *TPM1*, which showed both differential expression and isoform switching (see above), was a member of the green module (Fig. 2C,D; Table S3), further supporting its central role in the vascular response. Intramodular connectivity analysis identified a tightly connected network centered on five hub genes with the highest intramodular connectivity (kWithin): ADP-ribosylhydrolase-like 1 (*ADPRHL1*), Cardiomyopathy Associated 5 (*CMYA5*), LIM Domain Binding 3 (LDB3), Kelch-like Family Member 40 (*KLHL40*) and Titin (*TTN*). These genes are typically involved in cardiac and skeletal muscle cell function but are also expressed in the brain with roles in neuronal development and cytoskeletal structure (Blech-Hermoni et al., 2023; Nelson et al., 2021). These hub genes showed strong interactions (adjacency>0.4) with associated neighbor genes in the green module (Fig. 4B), suggesting orchestrated regulation of vascular contractile programs rather than independent cellular responses.

### Vascular gene expression changes localize to cerebrovascular cell types

**Fig. 5.**
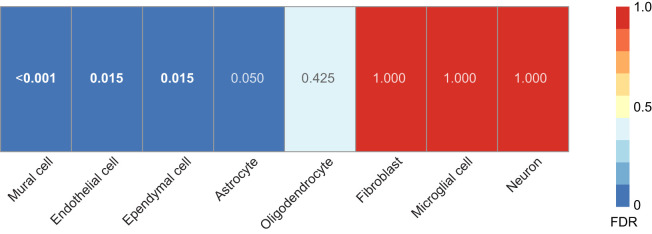
**Cell-type enrichment of the green WGCNA module using HYPOMAP markers.** Heatmap of FDR-corrected *P*-values from one-sided hypergeometric tests assessing overrepresentation of green WGCNA module genes (from *n*=19 hypothalamic samples: 10 heat call, 9 control; converted to human homologs via biomaRt) within HYPOMAP cell-type marker gene sets. Benjamini–Hochberg FDR correction applied across all module-by-cell-type tests. Dark blue indicates strong enrichment (bold indicates significance).

To identify the specific hypothalamic cell types in which gene expression changes occurred, we conducted cell-type enrichment analysis within each module (see Materials and Methods). The green module genes were significantly enriched in mural cells (VSMCs and pericytes; FDR<0.001), endothelial cells (FDR=0.015) and ependymal cells (FDR=0.015; Fig. 5), based on marker genes derived from a human hypothalamic single-cell atlas (HYPOMAP) (Tadross et al., 2025), as a comparable zebra finch embryo single-cell hypothalamic atlas is not yet available. These cell types collectively regulate cerebral perfusion, vascular barrier permeability and cerebrospinal fluid homeostasis, indicating that heat call exposure targets the integrated cerebrovascular system at the transcriptional level, rather than individual cell populations. This cell-type specificity is consistent with our secondary hypothesis that acoustic signals trigger localized cerebrovascular adaptations to prepare the brain for thermal stress. Genes in the brown and red modules showed no significant cell-type enrichment for hypothalamic cell types.

### Cellular composition of embryonic hypothalamic tissue

To estimate the cellular composition of bulk hypothalamic tissue and contextualize the magnitude of vascular cell responses, we employed CIBERSORTx deconvolution to derive cell type-specific relative fractions representing within-sample RNA contributions. Oligodendrocytes (median: 0.403 [interquartile range, IQR: 0.017]), neurons (median: 0.374 [IQR: 0.061]), astrocytes (median: 0.124 [IQR: 0.018]) and microglia (median: 0.071 [IQR: 0.010]) dominated the cellular milieu, collectively representing over 95% of the estimated RNA contribution (Fig. S4). Minor populations included mural cells (median: 0.014 [IQR: 0.015]), ependymal cells (median: 0.007 [IQR: 0.011]) and endothelial cells (median: 0.005 [IQR: 0.004]), with fibroblasts nearly absent (median: 0.000; mean: 0.001) (Fig. S4).

Across all samples, the CIBERSORTx deconvolution met stringent diagnostic criteria (goodness-of-fit *P*<0.0001, Pearson *r*>0.89; root mean square error, RMSE 0.70–0.75; Table S6). This cellular composition is consistent with parenchyma-enriched hypothalamic punches, confirming that the dissected tissue primarily captured neural and glial cell types rather than surrounding non-neural tissues. The high proportion of neurons and glia relative to vascular-associated cells (endothelial and mural cells comprising <2% of RNA contribution) indicates that transcriptional effects localized to vascular compartments would be substantially diluted in bulk RNA-Seq profiles. Despite their minimal RNA contribution (<2%), vascular-associated cells exhibited robust transcriptional responses to playback exposure (see ‘Co-expression network analysis reveals coordinated vascular gene regulation’, above), as evidenced by significant enrichment of the green WGCNA module in these cell types (Fig. 5). This pattern suggests that vascular cell populations undergo large expression changes that are detectable in bulk profiles despite their low abundance, consistent with targeted, cell type-specific transcriptional changes indicative of programming of cerebrovascular function.

## DISCUSSION

Developmental plasticity in response to environmental signals is widespread across taxa, but the physiological mechanisms linking sensory cues to specific cellular outcomes remain poorly understood. Our study reveals transcriptional modifications consistent with prenatal acoustic signals programming cerebrovascular function in the developing hypothalamus. Contrary to predictions of systemic neuroendocrine reprogramming, heat call exposure triggered coordinated downregulation of vascular contractile programs in endothelial cells, VSMCs and ependymal cells. Importantly, these changes occurred in response to an acoustic signal alone, without thermal exposure, demonstrating the potency of sensory-mediated developmental programming. To our knowledge, our study provides the first comprehensive transcriptomic characterization of the prenatal zebra finch hypothalamus and reveals a novel cellular mechanism for anticipatory thermal adaptation.

The cerebrovascular changes we observed involve an integrated vascular system composed of endothelial cells, VSMCs, pericytes, astrocytes and neurons that collectively regulate cerebral perfusion, barrier permeability and metabolic exchange (McConnell and Mishra, 2022). Endothelial cells form specialized junctions that control molecular exchange between the bloodstream and brain tissue (Atochin and Huang, 2011; Hogan-Cann et al., 2019; McConnell and Mishra, 2022; Stobart et al., 2013), while mural cells (VSMCs and pericytes) wrapped around cerebral blood vessels contribute to vascular barrier function and regulate blood flow through coordinated contraction and relaxation. VSMCs exhibit remarkable phenotypic plasticity in response to environmental cues, transitioning between a ‘contractile’ phenotype characterized by high expression of contractile proteins (smooth muscle actin, myosin heavy chain, tropomyosin) and a ‘synthetic’ phenotype with reduced contractile gene expression and elevated proliferation, migration and extracellular matrix production (Hayes et al., 2022; Vanlandewijck et al., 2018). Embryonic VSMCs normally exhibit a synthetic phenotype and mature to the contractile state in adulthood (Ball et al., 2010; Owens et al., 2004). Our results showing downregulation of contractile marker genes, including *TPM1*, a key regulator of the VSMC contractile phenotype (Li et al., 2025), as well as genes associated with cytoskeletal organization and cardiac muscle contraction pathways, suggest that heat call exposure may maintain or enhance the embryonic synthetic VSMC phenotype during prenatal hypothalamic development. This retention of the synthetic state could facilitate vascular remodeling and barrier adaptation in preparation for post-hatch thermal challenges.

Under normal temperature ranges, cerebral blood flow adjusts to temperature through temperature-sensing channels in VSMCs and endothelial cells that regulate Ca^2+^ influx and vascular tone (Bain et al., 2015; Hu and Zhang, 2024; Kuppusamy et al., 2021; Phan et al., 2025). However, whether endothermic brains possess specific defense mechanisms against hyperthermia remains unclear. In birds, the rete ophthalmicum functions as a cerebral heat exchanger but cools only a fraction of blood reaching the brain, with cooling efficiency remaining constant across temperatures (Arad and Midtgard, 1990; Bech and Midtgård, 1981; Jessen, 2001; Porter and Witmer, 2016). Similarly in humans, systemic thermoregulatory responses at high temperatures reduce cerebral blood flow, which decreases rather than enhances brain cooling (Bain et al., 2015). Under hyperthermia, heat-stress studies show breakdown of endothelial tight junctions mediated by pro-inflammatory molecules, leading to cerebral edema (Bain et al., 2015; Kiyatkin and Sharma, 2009; Yoneda et al., 2024). However, moderate loosening of barrier junctions during the initial stages of thermal challenges may prevent structural damage (Huber et al., 2001), suggesting that controlled vascular plasticity serves a protective function. Our findings showing downregulation of contractile and cytoskeletal programs in endothelial and mural cells likely represent such adaptive modifications. Importantly, because embryos were exposed to an acoustic signal rather than actual heat, the transcriptomic changes we observed are unlikely to reflect heat-induced damage, which typically involves upregulation of oxidative stress, mitochondrial dysfunction and inflammatory pathways (Fang et al., 2024; Yoneda et al., 2024). Investigating prenatal heat call effects therefore provides a unique opportunity to disentangle adaptive protective responses from detrimental cellular damage.

The downregulation of *TPM1* we observed may serve multiple functions in this context. First, reduced *TPM1* expression is associated with enhanced synthetic vascular smooth muscle phenotypes (Li et al., 2025), potentially facilitating vascular remodeling in preparation for thermal challenges. Second, in mammals, excessive *TPM1* triggers neuroinflammation through microglial activation (Li et al., 2022a,b), suggesting that *TPM1* downregulation may also function as an anticipatory anti-inflammatory modification. This is particularly relevant given that heat stress causes inflammatory damage in the brain (Yoneda et al., 2024) and may contribute to edema formation in zebra finch embryos experiencing hypoxia at high incubation temperatures (Choi et al., 2025). Microglia proliferation has recently been identified as an underlying mechanism for lifelong cerebrovascular programming following prenatal immune activation (Zhao et al., 2022), suggesting that early modulation of inflammatory pathways could have lasting effects on brain function. If these cerebrovascular modifications persist post-hatch, they could contribute to enhanced thermal tolerance in heat call-exposed offspring, potentially explaining the increased heat tolerance and altered thermal preferences previously observed in these birds as adults (Mariette and Buchanan, 2016; Pessato et al., 2022; Udino and Mariette, 2022; Udino et al., 2021).

The changes we observed were not immediate responses to playback (which stopped 15 h prior to sampling) but rather persistent transcriptional differences following multiple days of acoustic exposure, potentially analogous to an acclimatization period. We note that a recent commentary (Anttonen et al., 2025) has raised questions about whether laboratory playback volumes may exceed natural exposure levels. We acknowledge this uncertainty, although current measurements do not reflect embryonic experience (see Materials and Methods). In addition, the transcriptional changes we document are not consistent with a non-specific stress response: DEGs do not include canonical acute stress markers, and the pattern of gene expression is cell-type specific and consistent with preparatory vascular remodeling rather than generalized tissue damage. Furthermore, even though zebra finch embryos at E10–E13 may not have yet developed functional hearing, mechanoreceptors such as Herbst corpuscles in the developing beak, which are sensitive to vibroacoustic stimulation and present in late-stage avian embryos (Schneider et al., 2017; Toyoshima et al., 1992; Ziolkowski et al., 2022), may contribute to transduction of the acoustic signal through non-auditory pathways, representing an additional avenue for future investigation. Taken together, the persistence of cell-type specific transcriptional changes beyond the playback window is more consistent with a coordinated anticipatory response than with non-specific acoustic stress and is reminiscent of the transcriptional memory established during physiological acclimatization. In humans, heat tolerance and cerebrovascular resistance to thermal stress improve following heat acclimatization (Bain et al., 2015; Jeliazkova-Mecheva et al., 2006). Exposure to heat calls during prenatal life may therefore trigger defense mechanisms prior to heat exposure upon hatching. However, localized hypothalamic vascular changes are unlikely to fully account for organismal-level phenotypes documented in earlier studies, including altered growth trajectories, mitochondrial function and begging behavior (Mariette and Buchanan, 2016; Udino et al., 2021), indicating that additional systemic mechanisms developing post-hatch remain to be identified.

Contrary to our initial hypothesis, our findings provide limited support for neuroendocrine mechanisms as the primary driver of the heat-adapted phenotype observed in nestlings and adults. Targeted analyses of hypothalamic neuroendocrine genes fell below corrected significance thresholds, despite circumventing some limitations of multiple hypothesis testing. Consistent with this finding, heat call exposure reduced nestling heat-shock protein levels and heterophile-to-lymphocyte ratios under hot conditions but did not alter plasma corticosterone levels (Udino et al., 2024), arguing against systemic neuroendocrine control. Nonetheless, postnatal hypothalamic gene regulation should be directly investigated to test whether the modest neuroendocrine programming we detected in embryos may strengthen postnatally when phenotypic changes in growth and begging emerge in response to temperature (Mariette and Buchanan, 2016). Alternatively, non-hormonal pathways may orchestrate the postnatal heat-adapted phenotype. Changes in nestling mitochondrial function following heat call exposure point to metabolic reprogramming as one such pathway (Udino et al., 2021). Additionally, in adult zebra finches, acute heat challenge upregulates dopaminergic pathways in the telencephalon, with gene expression levels positively correlated with individual panting behavior (Lipshutz et al., 2022). As panting in adulthood varies with early-life exposure to heat calls (Udino and Mariette, 2022), dopaminergic programming may also shape the heat call-induced phenotype. Lastly, the localized vascular changes we observed could also in part contribute to some traits of the postnatal heat-adapted phenotype if they persist and reduce brain susceptibility to heat damage in the long term. Namely, they could contribute to the higher heat tolerance observed in heat call-exposed birds at adulthood, which was not explained by more efficient evaporative cooling (Pessato et al., 2022), as well as their preference for hotter microsites (Mariette and Buchanan, 2016).

Our findings reveal a cellular mechanism for thermal adaptation that may represent a conserved strategy across endotherms facing predictable thermal challenges. For example, long-term heat acclimation in rats involves cell-type specific plasticity in the hypothalamic ependymal zone (Matsuzaki et al., 2009); prenatal heat exposure programs vascular thermoregulation at maturity in cattle (Ahmed et al., 2017); and embryonic thermal manipulation in both chickens and Japanese quail produces lasting vascular gene expression changes and modulation of peripheral blood flow consistent with thermotolerance programming (Loyau et al., 2016; Vitorino Carvalho et al., 2020). The cell-type specificity of the transcriptional response we observed, localized to vascular endothelial cells, smooth muscle cells and ependymal cells that together comprise less than 2% of hypothalamic RNA content, suggests large-magnitude expression changes within these populations. This targeted response may be mechanistically advantageous: rather than broadly reprogramming the entire hypothalamus, heat call exposure appears to selectively modify the cellular systems most vulnerable to thermal stress. Such specificity could minimize potential developmental trade-offs while maximizing preparatory benefits. The coordinated nature of these changes across functionally related cell types, revealed by our network analysis, further suggests an orchestrated cerebrovascular remodeling program rather than independent cellular responses. Whether this coordination reflects direct cell–cell signaling or parallel responses to a common upstream signal remains to be determined.

In the context of accelerating climate change, understanding mechanisms of thermal adaptation is increasingly urgent. Heat-related mortality is rising in both wildlife and humans, with arid-adapted species such as zebra finches experiencing reproductive failure during extreme heat events (Albright et al., 2017; Conradie et al., 2020; Howard et al., 2024; Intergovernmental Panel on Climate Change, 2015; Narayanan and Keellings, 2025; Stillman, 2019; Ummenhofer and Meehl, 2017). Our finding that acoustic cues alter transcriptional programs in cerebrovascular cell types suggests that sensory-mediated plasticity may influence developmental trajectories relevant to thermal tolerance, though whether these transcriptional changes translate to functional outcomes remains to be determined. However, the adaptive value of this programming depends critically on whether parental signals accurately forecast conditions offspring will encounter, a match that may break down under rapidly changing climates. In the long term, zebra finches that were exposed to heat calls as embryos do not show elevated chronic stress at adulthood in cool conditions, relative to control call birds. This suggests that the developmental programming initiated by heat calls does not detectably compromise cold-weather resilience later in life (Udino et al., 2024). However, it remains to be established whether such programming would be detrimental were the weather no longer predictable in the short term, and if cold conditions were experienced during the nestling stage, when prenatal heat calls had instead forecast hot conditions. As heat calls are produced as a result of vocal panting that provides a direct thermoregulatory benefit to the calling parent, there may be limited scope for flexible signal production according to weather unpredictability (Mariette and Buchanan, 2019; Pessato et al., 2020). Beyond climate change adaptation, our findings extend the understanding of prenatal sensory influences on development more broadly. The cerebrovascular programming documented here is triggered by an auditory cue, yet vascular and circulatory gene expression programs appear to be a shared target of sensory programming across different modalities: in a recent study, we showed that light exposure drives gene expression changes in vascular and circulatory pathways of embryonic chick retina (Versace et al., 2022), suggesting that sensory-mediated vascular programming may be a general developmental mechanism across sensory modalities and vertebrate taxa.

The present study is only the first step in understanding the role of neurovascular programming by heat calls for heat adaptation. Gene expression in the embryonic hypothalamus was measured at a single post-exposure time point (E14), and follow-up sampling across postnatal stages will be required to determine whether these cerebrovascular transcriptional changes persist after hatching. In addition, because the hypothalamus can only be sampled once per individual and we prioritized the embryonic programming window, these expression patterns have not been directly correlated with individual thermal tolerance. The transcriptional changes we describe are therefore best understood as a molecular snapshot that is suggestive of a preparatory vascular remodeling program rather than a confirmed functional outcome. We note, however, that prior work in this system has demonstrated that prenatal heat call exposure produces clear whole-organism consequences, including improved heat tolerance and a preference for hotter conditions (Mariette and Buchanan, 2016; Pessato et al., 2022; Udino and Mariette, 2022). The cerebrovascular transcriptional program identified here provides a candidate molecular mechanism through which these particular organismal traits may be established, which future work should investigate. Although our deconvolution analysis confirmed that tissue punches were parenchyma enriched and identified the primary cellular targets of heat call-induced transcriptional changes, future integration of spatial transcriptomics with targeted histology would provide finer anatomical resolution of responsive transcripts within hypothalamic subregions.

Another consideration is sex: we observed pronounced transcriptomic sex differentiation in the embryonic hypothalamus (PC1: 48% variance; Fig. S2), indicating that sex is a major axis of variation at this developmental stage. Importantly, although sex ratios were imbalanced between playback groups, multiple lines of evidence argue against sex confounding the main playback conclusions. First, the modules most strongly associated with playback (green and red) showed no detectable association with sex (green: *r*=0.12, *P*=0.625; red: *r*=−0.04, *P*=0.856; Fig. 3), indicating qualitatively distinct axes of variation for sex versus playback. Second, the module most strongly associated with sex (brown; *r*=0.98, *P*<0.001; Fig. 3) showed an effect in the opposite direction to playback (downregulated in heat call embryos but upregulated in females), implying that the sex imbalance would tend to attenuate rather than inflate playback-associated differences. While we did not recover significant GO-term enrichment for the brown module, the strong module–sex association and the presence of sex-linked transcripts among sex-by-treatment interactions (e.g. Z-linked *HDHD2* and W-linked *LOC116806857* and *LOC116806879*/*KCMF1*) still support sex-biased regulation at this developmental stage. Consistent with this, future studies with balanced sex ratios are likely to improve power to detect additional playback-responsive transcripts and to more fully resolve sex-by-treatment interactions (*HDHD2*, *LOC116806857*, *LOC116806879*/*KCMF1*, *ABRACL*, *PTPRM*).

Overall, our findings demonstrate that an acoustic signal of impending heat stress triggers adaptive modifications to cerebrovascular cells in developing songbird embryos. The transcriptional changes we observed, including downregulation of contractile programs and shifts toward a more plastic vascular phenotype, are consistent with preparatory remodeling of cerebrovascular regulation that occurs in response to an acoustic signal alone (Bain et al., 2015; Boorman et al., 2023; Huber et al., 2001). Given the evolutionary conservation of cerebrovascular systems across vertebrates, the mechanisms described here may be broadly applicable. Our study opens research opportunities for investigating thermal adaptation mechanisms, sensory-mediated developmental programming and the comparative physiology of anticipatory plasticity across taxa facing predictable environmental challenges.

## Supplementary Material

10.1242/jexbio.252287_sup1 Supplementary information
