# Supplementary material for: Prenatal acoustic communication triggers adaptive vascular programming in the developing avian brain
Source: J Exp Biol. 2026 Jun 15;229(11):jeb252287. doi: 10.1242/jeb.252287 (PMC13327539; doi:10.1242/jeb.252287)
Supplement: Supplementary information [file jexbio-229-252287-s1.pdf]

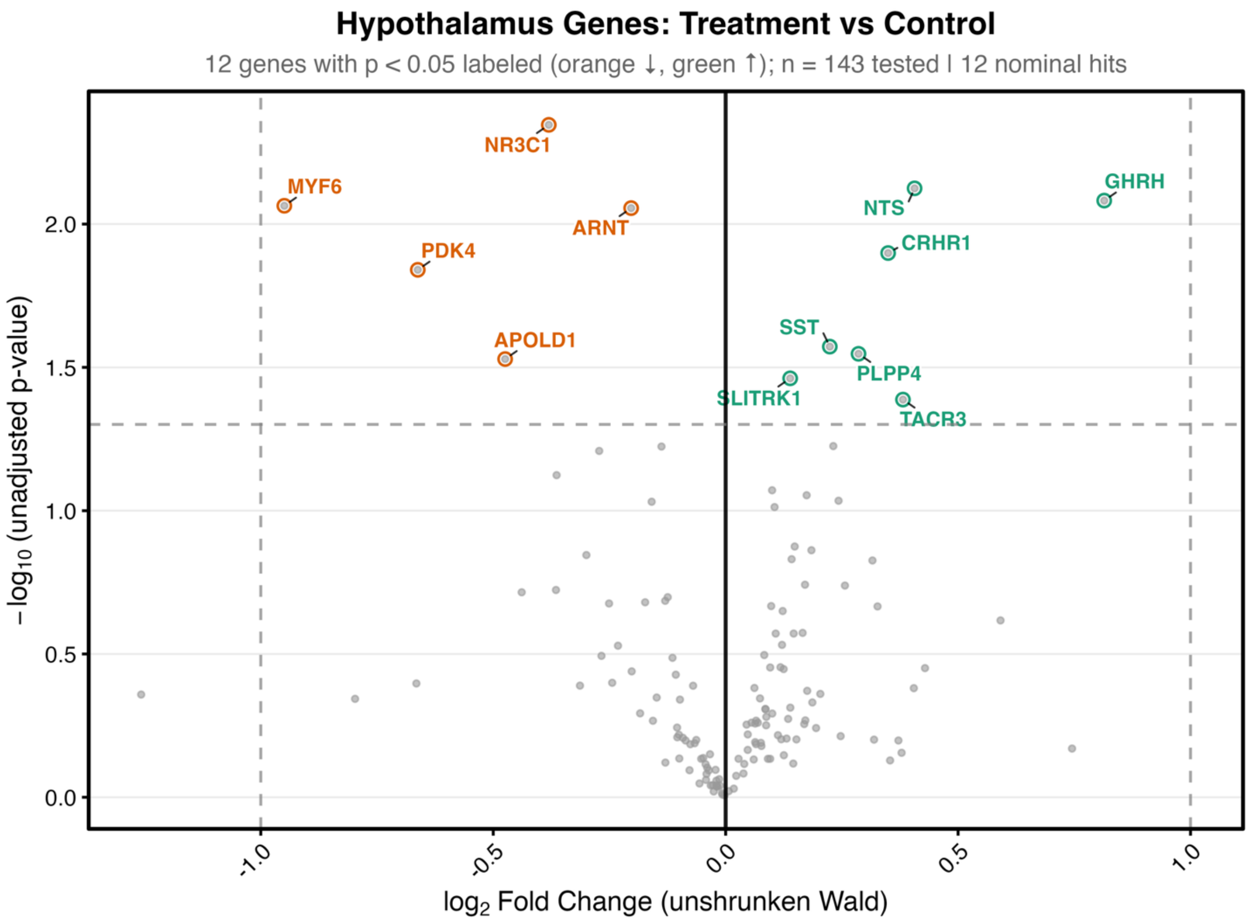

Fig. S1. Results of the Targeted Differential Gene Expression Analysis: Targeted differential gene expression analysis of 143 hypothalamic genes in medial hypothalamic punches (n=19: 10 heat call, 9 control embryos). Volcano plot shows log2 fold change (unshrunken Wald estimates; x-axis) versus −log10(unadjusted p-value; y-axis) from DESeq2. Twelve genes with nominal significance ($p < 0.05$, padj > 0.25) are highlighted: orange (downregulated with negative apeglm-shrunken log2FC: *NR3C1, MYF6, ARNT, PDK4, APOLD1*) and green (upregulated: *NTS, GHRH, CRHR1, SST, PLPP4, SLITRK1, TACR3*). Dashed lines: log2FC = ±1, p = 0.05.

Journal of Experimental Biology • Supplementary information

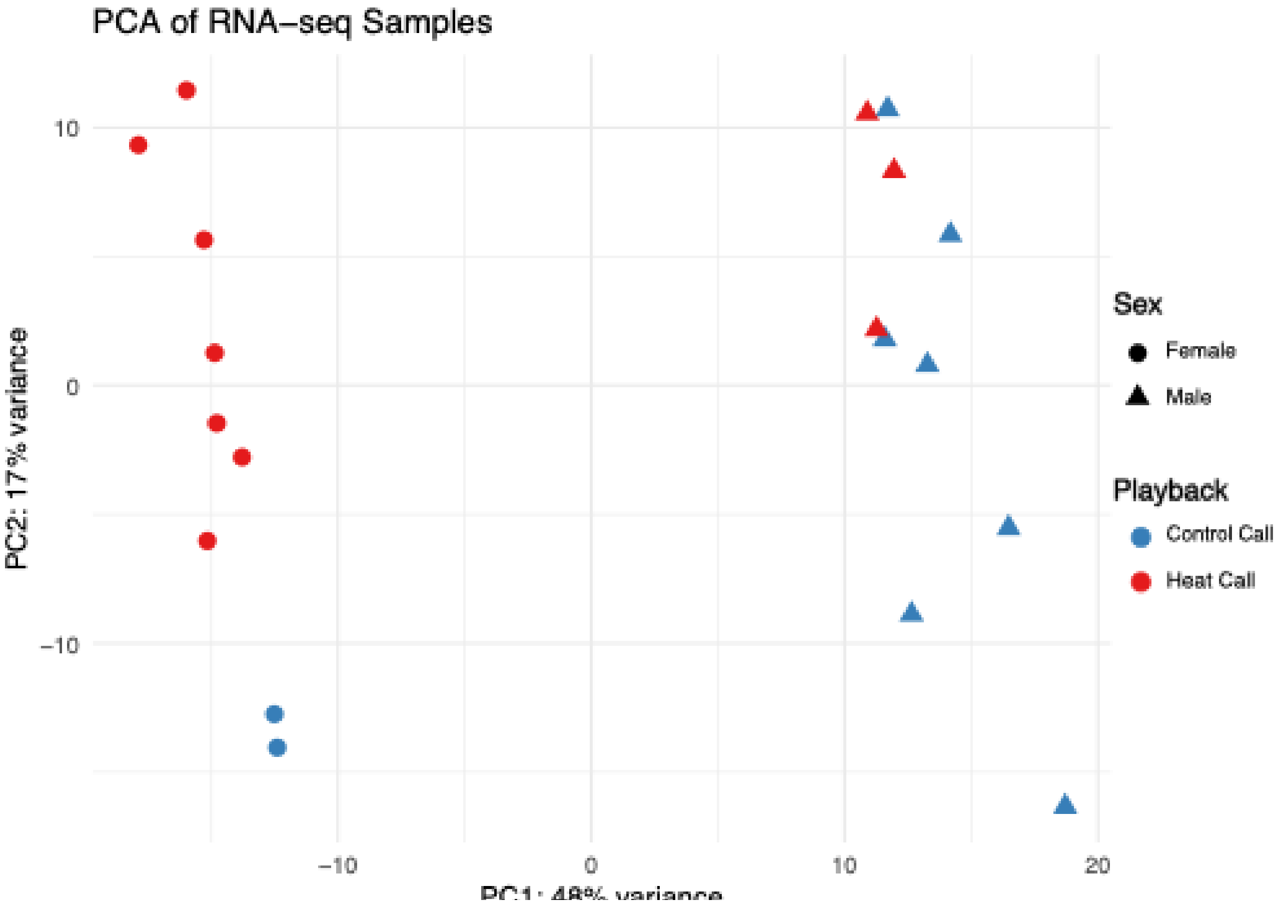

Fig. S2. Principal component analysis of variance-stabilizing transformed (VST) count data from medial hypothalamic punches (n=19 biological replicates: 10 heat call, 9 control). PC1 (48% variance) separates samples by sex; PC2 (17% variance) captures additional variation, including playback-associated separation Circles = female, triangles = male; red = heat call, blue = control call.

Journal of Experimental Biology • Supplementary information

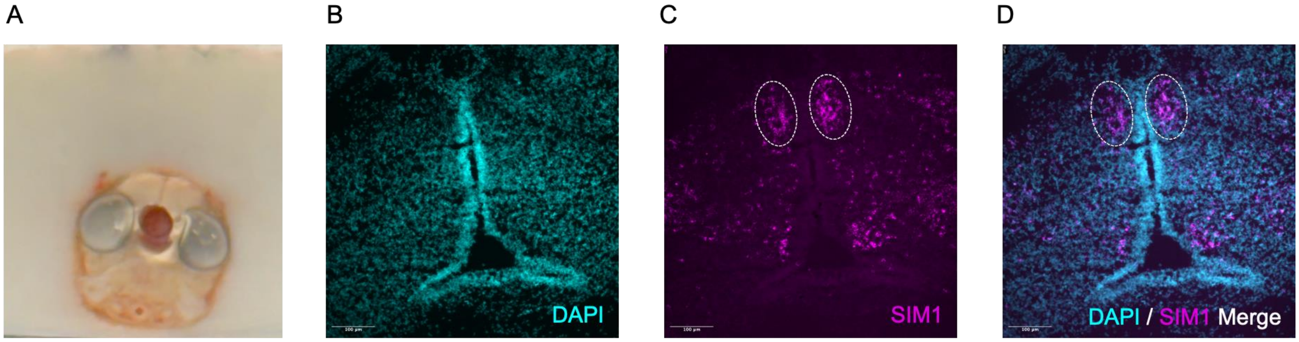

Fig. S3. (A) Medial hypothalamic punch encompassing the third ventricle and various hypothalamic nuclei from E13 Embryo. (B-D) Fluorescence in situ hybridization (FISH) pilot validation experiment of SIM BHLH Transcription Factor 1 (*SIM1*) expression in the developing hypothalamus. Representative image showing the hypothalamic marker gene *SIM1* mRNA signal in the (C) paraventricular nucleus of the hypothalamus (magenta), located lateral to the third ventricle, in an untreated embryo. (B and D) Nuclei are counterstained with DAPI (cyan). (C and D) Dotted lines delineate *SIM1*-positive cells within the paraventricular nucleus.

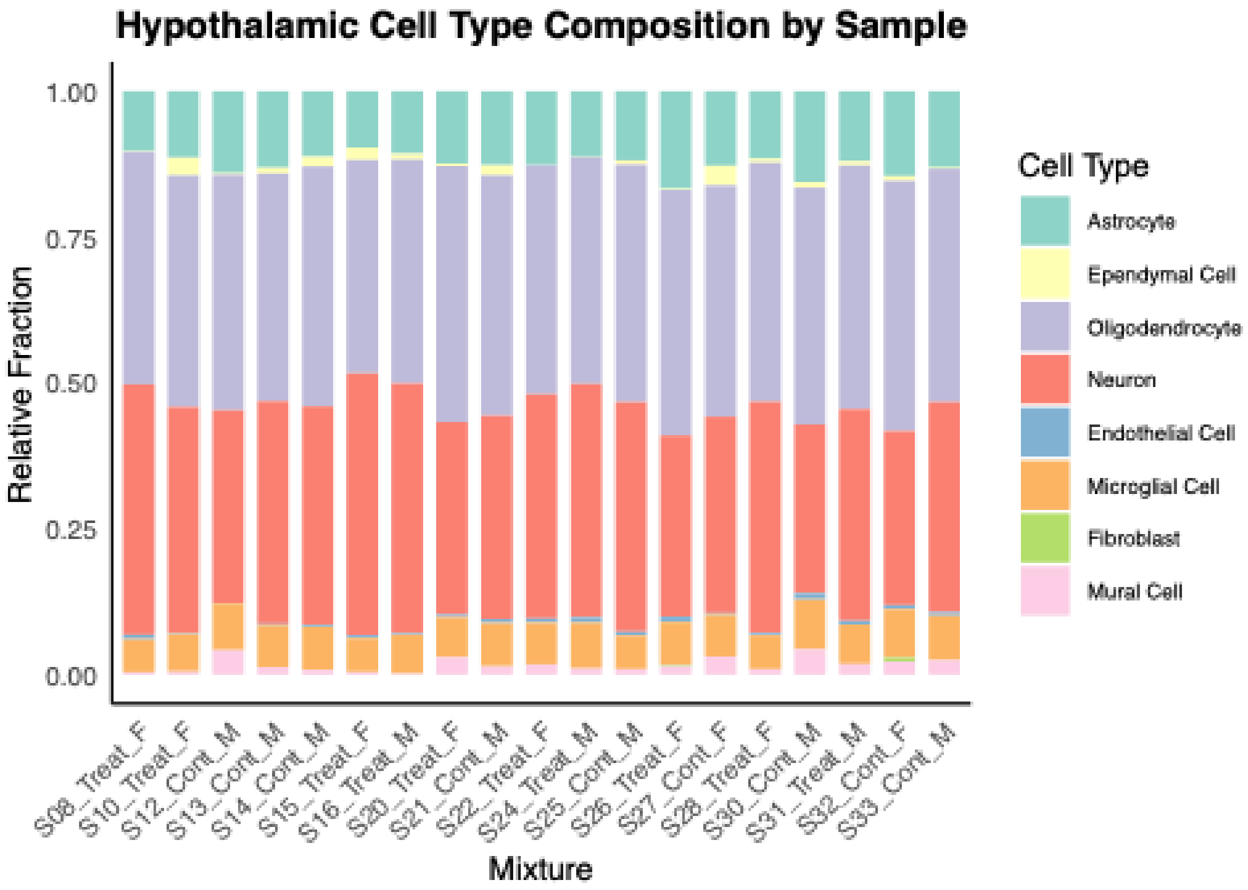

Fig. S4. CIBERSORTx deconvolution of bulk hypothalamic RNA-seq samples (n = 19: 10 heat call, 9 control; one medial punch per embryo). Stacked bars show estimated relative cell-type fractions (normalized to sum to 1) for eight brain cell types defined by the HYPOMAP human hypothalamus reference signature matrix. For each sample, CIBERSORTx reported a Monte Carlo permutation p-value (all samples $p < 0.0001$), the Pearson correlation coefficient between observed and model-predicted bulk expression restricted to signature genes ($r > 0.89$), and the root mean square error (RMSE; 0.70–0.75) between observed and model-predicted expression.

Journal of Experimental Biology • Supplementary information

Table S1. Hypothalamic Gene List for Targeted Analysis: Targeted differential gene expression results for 143 literature-derived hypothalamic genes in medial hypothalamic punches from heat call and control embryos.

Gene	Base Mean	Log2FC Unshrunken	LFC SE Unshrunken	Unadjusted p-value	FDR	Log2FC Shrunken apeglm	LFC SE Shrunken apeglm
NR3C1	1640.00	-0.38	0.13	**0.00**	0.25	-0.26	0.17
NTS	1204.06	0.41	0.15	**0.01**	0.25	0.05	0.10
GHRH	72.15	0.81	0.31	**0.01**	0.25	0.02	0.05
MYF6	88.39	-0.95	0.36	**0.01**	0.25	-0.02	0.05
ARNT	1125.44	-0.20	0.08	**0.01**	0.25	-0.13	0.09
CRHR1	744.52	0.35	0.14	**0.01**	0.29	0.07	0.15
PDK4	193.41	-0.66	0.27	**0.01**	0.29	-0.02	0.05
SST	5416.36	0.22	0.10	**0.03**	0.42	0.07	0.11
PLPP4	5652.55	0.29	0.13	**0.03**	0.42	0.05	0.09
APOLD1	164.51	-0.47	0.22	**0.03**	0.42	-0.02	0.05
SLITRK1	8001.38	0.14	0.07	**0.03**	0.45	0.07	0.07
TACR3	177.28	0.38	0.19	**0.04**	0.49	0.02	0.05
KCNA1	871.28	0.23	0.12	0.06	0.59	0.03	0.06
BMP7	1452.55	-0.14	0.07	0.06	0.59	-0.06	0.07
FOSL2	672.56	-0.27	0.15	0.06	0.59	-0.03	0.06
EMP1	666.97	-0.36	0.20	0.08	0.66	-0.02	0.05
ARPC5	19648.00	0.10	0.06	0.08	0.66	0.05	0.05
SLC10A4	1052.07	0.17	0.10	0.09	0.66	0.04	0.06
SLC18A3	1403.33	0.24	0.14	0.09	0.66	0.02	0.05
SLC2A1	2260.96	-0.16	0.09	0.09	0.66	-0.04	0.06
GCH1	1813.70	0.11	0.06	0.10	0.66	0.05	0.05
KCNJ6	690.93	0.15	0.10	0.13	0.82	0.03	0.05
PENK	8758.52	0.18	0.12	0.14	0.82	0.03	0.05
PTGS2	168.03	-0.30	0.20	0.14	0.82	-0.02	0.05
SIX3	2676.85	0.14	0.10	0.15	0.82	0.03	0.05

TRH	195.78	0.32	0.22	0.15	0.82	0.01	0.05
TAC1	3141.34	0.17	0.13	0.18	0.86	0.02	0.05
PVALB	1263.46	0.26	0.19	0.18	0.86	0.01	0.05
BMP4	219.36	-0.37	0.28	0.19	0.86	-0.01	0.05
MYH11	180.52	-0.44	0.34	0.19	0.86	-0.01	0.05
NOTCH1	2013.09	-0.12	0.10	0.20	0.86	-0.02	0.05
SGK1	2121.87	-0.13	0.10	0.21	0.86	-0.02	0.05
EDN3	386.01	-0.17	0.14	0.21	0.86	-0.02	0.05
THRA	778.78	-0.25	0.20	0.21	0.86	-0.01	0.05
SGSM1	1288.55	0.10	0.08	0.22	0.86	0.03	0.05
GAL	764.59	0.33	0.26	0.22	0.86	0.01	0.05
RASGRF2	757.89	0.12	0.10	0.22	0.87	0.02	0.05
LOC100219863	106.26	0.59	0.50	0.24	0.91	0.01	0.05
CBLN2	4674.73	0.17	0.15	0.27	0.91	0.01	0.05
NGB	345.51	0.15	0.13	0.27	0.91	0.01	0.05
ODC1	2999.70	0.11	0.10	0.27	0.91	0.02	0.05
TAGLN3	1765.27	0.12	0.12	0.29	0.91	0.02	0.05
DRD2	179.96	-0.23	0.22	0.30	0.91	-0.01	0.05
ATP2B2	7999.47	0.08	0.08	0.32	0.91	0.03	0.05
S100A10	1583.99	-0.27	0.27	0.32	0.91	-0.01	0.05
PTCH1	1941.38	-0.11	0.12	0.33	0.91	-0.02	0.05
CALB2	5086.41	0.12	0.13	0.35	0.91	0.01	0.05
ASCL1	421.67	0.10	0.10	0.35	0.91	0.02	0.05
NPVF	434.31	0.43	0.46	0.35	0.91	0.00	0.05
FGF10	216.93	0.12	0.14	0.36	0.91	0.01	0.05
SIX6	610.80	-0.20	0.22	0.36	0.91	-0.01	0.05
NUCB2	5085.61	-0.11	0.12	0.37	0.91	-0.01	0.04
HES5	192.74	-0.24	0.29	0.40	0.91	-0.01	0.05
NEUROD4	10.16	-0.67	0.79	0.40	0.91	-0.00	0.05
SLC38A8	87.44	-0.31	0.38	0.41	0.91	-0.00	0.05

Journal of Experimental Biology • Supplementary information

IGF1R	4671.41	-0.07	0.08	0.41	0.91	-0.02	0.04
SDC3	18110.13	0.06	0.08	0.42	0.91	0.02	0.04
AVPI1	66.14	0.40	0.50	0.42	0.91	0.00	0.05
LOC100231196	1935.95	0.18	0.22	0.43	0.91	0.01	0.05
HDC	103.68	0.20	0.26	0.44	0.91	0.01	0.05
UTS2	1.08	-1.26	1.62	0.44	0.91	-0.00	0.05
BMP3	1637.49	-0.15	0.20	0.45	0.91	-0.01	0.05
CELF6	3477.58	0.07	0.10	0.45	0.91	0.01	0.04
LOC115491955	1.44	-0.80	1.06	0.45	0.91	-0.00	0.05
BMP2	154.66	-0.10	0.13	0.46	0.91	-0.01	0.05
TCF7L2	4662.80	0.19	0.26	0.47	0.91	0.00	0.05
CRH	321.35	0.14	0.20	0.49	0.91	0.01	0.05
TRHDE	2164.43	0.09	0.12	0.49	0.91	0.01	0.04
SCG3	15178.33	0.09	0.13	0.49	0.91	0.01	0.04
AOAH	47.35	-0.18	0.28	0.51	0.91	-0.01	0.05
SNCG	2783.04	0.10	0.15	0.51	0.91	0.01	0.05
SLC18A2	1235.41	0.09	0.14	0.52	0.91	0.01	0.04
NKX2-1	812.18	0.13	0.22	0.53	0.91	0.01	0.05
TRHR	209.91	0.17	0.28	0.54	0.91	0.00	0.05
FABP7	95641.52	0.07	0.11	0.54	0.91	0.01	0.04
CHRDL1	717.28	-0.16	0.26	0.54	0.91	-0.01	0.05
SSTR1	7232.16	0.06	0.09	0.55	0.91	0.01	0.04
RXFP3	533.87	0.07	0.12	0.55	0.91	0.01	0.04
PGRMC2	2536.35	0.06	0.11	0.55	0.91	0.01	0.04
PRSS56	31.00	0.17	0.29	0.56	0.91	0.00	0.05
LZTS3	2261.80	0.04	0.08	0.56	0.91	0.01	0.04
CRHR2	1030.89	0.09	0.15	0.56	0.91	0.01	0.04
ATP2A3	711.20	-0.10	0.18	0.57	0.91	-0.01	0.05
POU3F1	149.97	0.19	0.35	0.57	0.91	0.00	0.05
IRS4	1706.76	0.05	0.09	0.60	0.91	0.01	0.04
IGF1	437.02	-0.10	0.19	0.61	0.91	-0.01	0.05

Journal of Experimental Biology • Supplementary information

RNF157	4351.07	0.11	0.22	0.61	0.91	0.00	0.05
MOV10L1	58.69	0.25	0.49	0.61	0.91	0.00	0.05
CALCB	886.12	-0.10	0.21	0.62	0.91	-0.01	0.05
TBX2	481.88	-0.09	0.19	0.62	0.91	-0.01	0.05
CARTPT	761.33	0.13	0.27	0.62	0.91	0.00	0.05
SYF2	3449.48	0.12	0.25	0.63	0.91	0.00	0.05
TSHR	163.93	0.15	0.31	0.63	0.91	0.00	0.05
LOC121469849	87.79	0.32	0.66	0.63	0.91	0.00	0.05
SSTR5	339.33	-0.06	0.13	0.63	0.91	-0.01	0.04
AVPR1B	12.75	0.37	0.78	0.63	0.91	0.00	0.05
MEST	227.01	-0.09	0.18	0.63	0.91	-0.01	0.05
INSIG1	3892.42	0.06	0.14	0.64	0.91	0.01	0.04
ROBO2	5056.93	0.08	0.16	0.65	0.91	0.01	0.04
NR3C2	640.71	-0.07	0.15	0.65	0.91	-0.01	0.04
MEIS1	3783.81	0.07	0.14	0.65	0.91	0.01	0.04
DLK1	1271.10	-0.08	0.17	0.65	0.91	-0.00	0.04
HMOX1	1528.92	0.08	0.18	0.66	0.91	0.01	0.04
PPP1R17	2.00	0.75	1.78	0.68	0.91	0.00	0.05
SHH	1995.73	0.05	0.12	0.68	0.91	0.01	0.04
CORT	3.10	0.38	0.98	0.70	0.91	0.00	0.05
DLL1	868.77	-0.03	0.09	0.71	0.91	-0.01	0.04
CHGA	19735.64	0.13	0.34	0.71	0.91	0.00	0.05
SCGN	415.02	-0.05	0.14	0.73	0.91	-0.01	0.04
OXTR	153.68	-0.10	0.29	0.73	0.91	-0.00	0.05
ERP29	11960.98	0.03	0.08	0.73	0.91	0.01	0.04
PDYN	501.78	0.10	0.28	0.73	0.91	0.00	0.05
ARHGAP35	2783.63	-0.05	0.15	0.73	0.91	-0.00	0.04
SSTR4	575.92	0.09	0.27	0.73	0.91	0.00	0.05
LHX5	1669.55	0.06	0.18	0.74	0.91	-0.00	0.04
NHLH1	4.63	0.35	1.08	0.74	0.91	0.00	0.05

Journal of Experimental Biology • Supplementary information

POMC	1098.31	-0.13	0.42	0.76	0.91	-0.00	0.05
SIM2	44.89	0.15	0.48	0.76	0.91	0.00	0.05
KCNIP2	2067.67	0.04	0.13	0.77	0.91	0.00	0.04
TH	540.42	-0.04	0.15	0.77	0.91	-0.00	0.04
AVPR1A	365.60	-0.04	0.15	0.79	0.93	-0.00	0.04
TRIP4	893.79	-0.02	0.09	0.80	0.93	-0.00	0.04
HMX2	153.46	-0.08	0.31	0.80	0.93	-0.00	0.05
SLC6A6	5000.41	-0.04	0.15	0.81	0.93	-0.00	0.04
SLC29A4	1555.32	0.04	0.17	0.83	0.94	0.00	0.04
SSTR2	1650.08	-0.04	0.19	0.83	0.94	-0.00	0.04
DBI	9375.89	0.02	0.11	0.84	0.95	0.00	0.04
HEY1	1259.53	-0.01	0.08	0.86	0.96	-0.00	0.04
SOX14	1572.19	-0.04	0.26	0.87	0.96	-0.00	0.05
THRB	625.49	-0.02	0.13	0.87	0.96	-0.00	0.04
LOC121469847	824.27	-0.06	0.43	0.89	0.96	-0.00	0.05
KCNMB4	130.27	-0.02	0.17	0.91	0.96	-0.00	0.04
FEZF1	958.21	-0.03	0.24	0.91	0.96	-0.00	0.05
SIM1	124.53	-0.03	0.28	0.91	0.96	-0.00	0.05
SLC17A6	2930.37	-0.01	0.11	0.91	0.96	-0.00	0.04
SLIT1	12650.29	-0.02	0.17	0.91	0.96	-0.00	0.04
ADCYAP1R1	710.41	-0.02	0.18	0.92	0.96	-0.00	0.04
ECEL1	1127.51	0.02	0.21	0.93	0.97	0.00	0.04
CALCR	696.66	0.01	0.11	0.95	0.97	0.00	0.04
PMCH	59.33	-0.03	0.45	0.95	0.97	-0.00	0.05
HMX3	1714.50	-0.01	0.19	0.97	0.98	-0.00	0.04
EMX2	917.69	-0.01	0.26	0.98	0.98	-0.00	0.05
CPNE2	1755.28	-0.00	0.11	0.98	0.98	-0.00	0.04

Journal of Experimental Biology • Supplementary information

Table S2. Genome-wide differential gene expression in medial hypothalamic punch RNA-seq from heat call-programmed zebra finches.

Gene	Base Mean	Log2FoldChange	lfcSE	Stat	pvalue	padj
LOC115493855	102.92	-1.97	0.37	28.24	1.07E-07	1.93E-03
SLC25A4	2833.02	-1.29	0.26	25.06	5.55E-07	3.37E-03
TNNC2	8193.28	-1.83	0.36	25.04	5.61E-07	3.37E-03
DHRS7C	142.13	-1.44	0.32	20.32	6.56E-06	2.36E-02
KLHL31	465.20	-1.79	0.40	20.44	6.15E-06	2.36E-02
ACTA1	25842.32	-1.88	0.43	18.79	1.46E-05	2.45E-02
FAM180B	5.82	-3.19	0.71	19.12	1.23E-05	2.45E-02
FBXL22	223.41	-1.39	0.33	18.58	1.63E-05	2.45E-02
KLHL40	498.57	-1.56	0.36	19.30	1.12E-05	2.45E-02
LDB3	2132.07	-1.59	0.36	19.18	1.19E-05	2.45E-02
LOC115495486	137.86	-1.97	0.48	19.11	1.23E-05	2.45E-02
MUSTN1	803.68	-1.62	0.37	18.68	1.55E-05	2.45E-02
MYOM1	1367.09	-1.50	0.36	18.27	1.92E-05	2.6E-02
PACSIN3	1502.59	-1.13	0.27	18.17	2.02E-05	2.6E-02
CMYA5	390.52	-1.52	0.37	18.02	2.18E-05	2.62E-02
MYOT	1360.14	-1.54	0.36	17.81	2.45E-05	2.75E-02
FSD2	154.26	-1.35	0.33	17.51	2.86E-05	2.83E-02
LOC101233779	123.12	-1.34	0.32	17.43	2.99E-05	2.83E-02
NEB	2167.99	-1.77	0.43	17.47	2.92E-05	2.83E-02
CLIC5	70.57	-1.16	0.29	17.25	3.28E-05	2.96E-02
OBSCN	878.09	-1.36	0.34	17.02	3.7E-05	3.17E-02
ATP1B4	347.24	-1.29	0.32	16.90	3.95E-05	3.23E-02
CASQ2	2358.59	-1.73	0.43	16.63	4.55E-05	3.5E-02
LOC105758604	7951.80	0.20	0.05	16.43	5.06E-05	3.5E-02
LOC115493912	38.21	-1.54	0.41	16.52	4.81E-05	3.5E-02

LOC121468823	15.09	-1.80	0.44	16.46	4.96E-05	3.5E-02
TNNT3	23912.25	-1.43	0.36	15.88	6.73E-05	4.37E-02
TRIM55	170.78	-1.48	0.39	15.87	6.8E-05	4.37E-02
UNC45B	515.04	-1.32	0.34	15.76	7.2E-05	4.39E-02
ZNF106	2405.54	-0.79	0.21	15.73	7.32E-05	4.39E-02
MYLK2	213.82	-1.43	0.39	15.53	8.1E-05	4.45E-02
MYOZ1	442.64	-1.95	0.50	15.52	8.16E-05	4.45E-02
TNNI2	4836.21	-1.69	0.43	15.58	7.9E-05	4.45E-02
ASB12	238.01	-1.38	0.36	15.33	9.04E-05	4.65E-02
PPP1R3A	179.42	-1.40	0.37	15.36	8.89E-05	4.65E-02
LOC100218875	2095.71	-1.81	0.49	15.18	9.79E-05	4.67E-02
SLC22A16	141.68	-1.25	0.34	15.19	9.7E-05	4.67E-02
XIRP1	591.38	-1.56	0.41	15.11	1.01E-04	4.67E-02
YIPF7	54.77	-0.85	0.22	15.13	1E-04	4.67E-02
FHL1	2000.44	-1.78	0.45	15.06	1.04E-04	4.69E-02
SRL	2776.20	-1.38	0.37	14.98	1.09E-04	4.78E-02
CACNG1	755.42	-1.35	0.35	14.84	1.17E-04	4.81E-02
EMILIN3	680.55	-0.50	0.13	14.71	1.26E-04	4.81E-02
LOC100228485	1664.12	-2.32	0.56	14.87	1.15E-04	4.81E-02
LOC100230937	32.60	-1.45	0.39	14.77	1.21E-04	4.81E-02
LOC105760992	29574.71	-1.93	0.50	14.71	1.25E-04	4.81E-02
TPM2	16134.92	-1.06	0.28	14.82	1.19E-04	4.81E-02
LOC115495214	50.13	-1.77	0.47	14.64	1.3E-04	4.88E-02
DES	2910.36	-1.43	0.36	14.57	1.35E-04	4.95E-02

Table S3. Gene Ontology Biological Process enrichment results for modules correlated with heat call playback. Green, brown, and red module genes were analyzed using ShinyGO (v0.85, zebra finch) using hypergeometric tests with Benjamini–Hochberg FDR correction. Number of genes, fold enrichment, and a list of enriched genes are provided for each significant GO term (the brown module did not return significant GO term).

Pathway	Enrichment FDR	Module	Number of Genes	Pathway Genes	Fold Enrichment	Genes
Path:hsa04820 Cytoskeleton in muscle cells	2.64E-33	Green	48	232	10.21	*PDLIM5, LDB3, MYOM3, COL6A2, DES, SYNPO2, FHL1, ATP1B4, ANKRD2, AMPD1, PDLIM3, LMNA, LMOD2, MYBPC1, MYBPC3, MYL1, MYL2, MYL3, NEB, MYOZ2, LMOD3, ACTA1, MYOZ1, SGCD, SGCG, SNTB1, ACTC1, TMOD1, TNNC2, TNNC1, TNNI1, TNNI2, TNNT2, TNNT3, TPM1, TPM2, TPM4, VIM, CSRP3, SSPN, CAPN3, MYPN, TRIM55, MYOM1, ACTN2, PDLIM1, MYOM2, MYOT*
Path:hsa04260 Cardiac muscle contraction	5.74E-06	Green	11	87	7.83	*ATP1B4, MYL2, MYL3, ACTC1, TNNC1, TNNT2, TPM1, TPM2, TPM4, CACNG1, CASQ2*
Path:hsa05410 Hypertrophic cardiomyopathy	8.89E-09	Green	17	99	7.42	*DES, LMNA, MYBPC3, MYL2, MYL3, SGCD, SGCG, SNTB1, ACTC1, TNNC1, TNNT2, TPM1, TPM2, TPM4, CACNG1, SSPN, CAV3*
Path:hsa05414 Dilated cardiomyopathy	1.10E-08	Green	17	105	7.15	*DES, LMNA, MYBPC3, MYL2, MYL3, SGCD, SGCG, SNTB1, ACTC1, TNNC1, TNNT2, TPM1, TPM2, TPM4, CACNG1, SSPN, CAV3*
Path:hsa05416 Viral myocarditis	1.23E-02	Green	6	69	5.98	*SGCD, SGCG, SNTB1, SSPN, CAV1, CAV3*
Path:hsa04814 Motor proteins	1.73E-05	Green	16	194	4.58	*DYNC1I1, MYL1, MYL2, MYL3, MYO5C, ACTA1, ACTC1, TNNC2, TNNC1, TNNI1, TNNI2, TNNT2, TNNT3, TPM1, TPM2, TPM4*
Path:hsa05412 Arrhythmogenic right ventricular cardiomyopathy	5.37E-03	Green	9	86	4.49	*DES, LMNA, SGCD, SGCG, SNTB1, CACNG1, SSPN, CAV3, ACTN2*
Path:hsa04261 Adrenergic signaling in cardiomyocytes	5.23E-04	Green	13	153	4.16	*BVES, AGTR1, ATP1B4, MYL2, MYL3, POPDC2, ACTC1, TNNC1, TNNT2, TPM1, TPM2, TPM4, CACNG1*
GO:0006936 Muscle contraction	4.25E-02	Red	19	360	2.91	*CHRM2, SRF, ATP2A1, MAP2K3, ARG2, CACNA1S, CHRND, ATP1B1, TRIM63, ATP1A1, ABAT, ANXA6, SNTA1, FGF12, NMUR2, CRYAB, GAMT, MYBPH, CHRNG*
GO:0003012 Muscle system process	2.35E-02	Red	23	450	2.75	*CHRM2, SRF, ATP2A1, MAP2K3, ACACB, ARG2, CACNA1S, MYOG, HEY2, CHRND, ATP1B1, TRIM63, ATP1A1, ABAT, MYMK, ANXA6, SNTA1, FGF12, NMUR2, CRYAB, GAMT, MYBPH, CHRNG*
GO:0061061 Muscle structure development	1.69E-02	Red	33	721	2.35	*MYF5, MYOG, PDLIM4, MYBPH, ALPK3, DOCK1, PDGFB, MYMK, SOD2, EHD2, YBX3, CACNA1S, CRYAB, SRF, MEF2D, TCF21, WFIKKN1, FLNC, HOXD9, AKIRIN2, HEY2, SYPL2, UCHL1, FBXO40, HSPB2, TMEM119, PPP2R3A, CHRND, BOC, OBSL1, CHODL, ITGB1BP2, FHL3*

Table S4. Significant isoform switches (FDR < 0.05, differential isoform fraction ≥ 0.05) in medial hypothalamic RNA-seq from embryos exposed to chronic heat call versus control playback.

Isoform ID	Gene Symbol	Reference Condition	Treatment Condition	Isoform Fraction in Control	Isoform Fraction in Heat Call	Change in isoform fraction (ΔIF)	q-value
XM_041721404.1	*TLK2*	cont	treat	0.06	0	-0.06	9.4E-07
XM_041714671.1	*LOC100228270*	cont	treat	0.33	0.07	-0.26	8.6E-03
NM_001136481.2	*TPM1*	cont	treat	0.66	0.43	-0.23	8.6E-03
XM_030265025.3	*TCAIM*	cont	treat	0.17	0.02	-0.15	1.2E-02
XM_030285413.3	*EIF4ENIF1*	cont	treat	0.21	0.38	0.17	1.6E-02
XM_041721341.1	*CELSR2*	cont	treat	0.78	0.58	-0.2	2.4E-02
XM_030269705.3	*GPCPD1*	cont	treat	0.44	0.64	0.21	2.4E-02
XM_041721627.1	*MPV17L2*	cont	treat	0.03	0.39	0.36	2.4E-02
XM_041721628.1	*MPV17L2*	cont	treat	0.97	0.61	-0.36	2.4E-02
XM_030272879.3	*RLIM*	cont	treat	0.71	0.88	0.17	2.4E-02
XM_030272880.3	*RLIM*	cont	treat	0.29	0.1	-0.18	2.4E-02
XM_002191938.4	*TSPEAR*	cont	treat	0.84	0.94	0.1	2.4E-02
XM_041717575.1	*CARF*	cont	treat	0.08	0.02	-0.07	3E-02
XM_030280110.3	*ARHGEF7*	cont	treat	0.16	0.04	-0.12	3.3E-02
XM_030286601.3	*RGS3*	cont	treat	0.08	0.16	0.07	3.3E-02
XM_030280436.3	*TSPEAR*	cont	treat	0.16	0.06	-0.1	3.3E-02
XM_030266254.3	*LOC121468029*	cont	treat	0.27	0.14	-0.13	3.6E-02
XM_030271401.3	*CBR4*	cont	treat	0.07	0.01	-0.06	3.9E-02
XM_030263200.3	*CHPT1*	cont	treat	0.11	0.05	-0.06	3.9E-02
XM_030284686.3	*GSG1L*	cont	treat	0.16	0.05	-0.11	3.9E-02
XM_030267870.3	*PTPRK*	cont	treat	0.34	0.55	0.21	3.9E-02
XM_012573306.4	*TBC1D9*	cont	treat	0.28	0.16	-0.12	3.9E-02
XM_030271911.3	*TBC1D9*	cont	treat	0.72	0.84	0.12	3.9E-02
XM_002200039.6	*ARFGAP2*	cont	treat	0.91	0.98	0.07	3.9E-02
XM_030283390.3	*DENND6A*	cont	treat	0	0.24	0.24	3.9E-02
XM_030275365.3	*LOC100222881*	cont	treat	0.11	0.4	0.29	3.9E-02
XM_030275366.3	*LOC100222881*	cont	treat	0.89	0.6	-0.29	3.9E-02
XM_030274227.3	*ARFGAP2*	cont	treat	0.09	0.02	-0.07	4E-02

Journal of Experimental Biology • Supplementary information

**XM_002189844.6**	*DHTKD1*	cont	treat	0.9	0.73	-0.17	4.4E-02
**XM_032751614.2**	*RAB37*	cont	treat	0.42	0.08	-0.34	4.4E-02
**XM_041717123.1**	*LOC121470179*	cont	treat	0.24	0.01	-0.23	4.7E-02
**XM_030290503.3**	*DHTKD1*	cont	treat	0.1	0.27	0.17	4.9E-02
**XM_030288176.3**	*ANKFY1*	cont	treat	0.45	0.25	-0.2	4.9E-02
**XM_030288177.3**	*ANKFY1*	cont	treat	0.55	0.75	0.2	4.9E-02

Table S5. Alternative splicing event enrichment among significant isoform switches in medial hypothalamic RNA-seq from heat call and control embryos.

Splicing Type	Number of Gains in Heat Call	Number of Losses in Heat Call	Total Events	Proportion of Gains	P-value	q-value
ES (Exon Skipping)	4	13	17	0.24	0.049	0.34
A3SS (Alt 3' Splice Site)	5	1	6	0.83	0.219	0.77
A5SS (Alt 5' Splice Site)	0	1	1	0	1.000	1.00
ATSS (Alt Transcription Start)	6	3	9	0.67	0.508	1.00
ATTS (Alt Transcription Term)	3	2	5	0.6	1.000	1.00
IR (Intron Retention)	0	1	1	0	1	1
MES (Mutually Exclusive Exons)	3	2	5	0.6	1.000	1.00

Table S6. CIBERSORTx absolute-mode cell-type scores and deconvolution diagnostics for bulk hypothalamic RNA-seq samples.

Mixture	astrocyte	ependymal cell	oligodendrocyte	neuron	endothelial cell	microglial cell	fibroblast	mural cell	P-value	Correlation	RMSE	Absolute score (sig.score)
S08_Treat_F	0.180	0.002	0.692	0.746	0.013	0.098	0.000	0.007	0.000	0.977	0.700	1.738
S10_Treat_F	0.193	0.052	0.673	0.658	0.003	0.109	0.000	0.008	0.000	0.894	0.749	1.698
S12_Cont_M	0.238	0.005	0.683	0.559	0.001	0.136	0.000	0.071	0.000	0.914	0.750	1.693
S13_Cont_M	0.224	0.017	0.665	0.650	0.004	0.122	0.000	0.022	0.000	0.962	0.721	1.704

Journal of Experimental Biology • Supplementary information

**S14_Cont_M**	0.189	0.028	0.691	0.628	0.006	0.125	0.000	0.013	0.000	0.974	0.719	1.680
**S15_Treat_F**	0.173	0.039	0.655	0.805	0.009	0.104	0.000	0.008	0.000	0.903	0.732	1.792
**S16_Treat_M**	0.192	0.017	0.682	0.762	0.005	0.118	0.000	0.003	0.000	0.968	0.707	1.780
**S20_Treat_F**	0.207	0.007	0.730	0.551	0.007	0.114	0.000	0.050	0.000	0.898	0.754	1.665
**S21_Cont_M**	0.218	0.030	0.706	0.598	0.012	0.127	0.000	0.024	0.000	0.956	0.731	1.715
**S22_Treat_F**	0.216	0.000	0.669	0.654	0.012	0.121	0.000	0.030	0.000	0.981	0.711	1.703
**S24_Treat_M**	0.192	0.001	0.659	0.679	0.016	0.134	0.000	0.017	0.000	0.975	0.711	1.698
**S25_Cont_M**	0.200	0.012	0.684	0.661	0.011	0.097	0.000	0.015	0.000	0.972	0.711	1.680
**S26_Treat_F**	0.292	0.004	0.734	0.541	0.017	0.126	0.005	0.025	0.000	0.953	0.735	1.744
**S27_Cont_F**	0.215	0.057	0.665	0.565	0.004	0.119	0.000	0.052	0.000	0.961	0.734	1.678
**S28_Treat_F**	0.197	0.011	0.695	0.672	0.007	0.099	0.000	0.015	0.000	0.969	0.712	1.695
**S30_Cont_M**	0.260	0.016	0.675	0.481	0.018	0.142	0.000	0.072	0.000	0.939	0.749	1.663
**S31_Treat_M**	0.204	0.011	0.706	0.613	0.010	0.114	0.000	0.031	0.000	0.974	0.719	1.689
**S32_Cont_F**	0.253	0.014	0.742	0.517	0.011	0.144	0.012	0.038	0.000	0.955	0.735	1.731
**S33_Cont_M**	0.221	0.004	0.671	0.604	0.009	0.129	0.000	0.041	0.000	0.977	0.719	1.678

References

Choi, H. M. T., Beck, V. A., & Pierce, N. A. (2014). Next-generation in situ hybridization chain reaction: higher gain, lower cost, greater durability. *ACS Nano*, *8*(5), 4284–4294.

Choi, H. M. T., Calvert, C. R., Husain, N., Huss, D., Barsi, J. C., Deverman, B. E., Hunter, R. C., Kato, M., Lee, S. M., Abelin, A. C. T., Rosenthal, A. Z., Akbari, O. S., Li, Y., Hay, B. A., Sternberg, P. W., Patterson, P. H., Davidson, E. H., Mazmanian, S. K., Prober, D. A., … Pierce, N. A. (2016). Mapping a multiplexed zoo of mRNA expression. *Development (Cambridge, England)*, *143*(19), 3632–3637.

Choi, H. M. T., Chang, J. Y., Trinh, L. A., Padilla, J. E., Fraser, S. E., & Pierce, N. A. (2010). Programmable in situ amplification for multiplexed imaging of mRNA expression. *Nature Biotechnology*, *28*(11), 1208–1212.

Choi, H. M. T., Schwarzkopf, M., Fornace, M. E., Acharya, A., Artavanis, G., Stegmaier, J., Cunha, A., & Pierce, N. A. (2018). Third-generation in situ hybridization chain reaction: multiplexed, quantitative, sensitive, versatile, robust. *Development (Cambridge, England)*, *145*(12). https://doi.org/10.1242/dev.165753

Schindelin, J., Arganda-Carreras, I., Frise, E., Kaynig, V., Longair, M., Pietzsch, T., Preibisch, S., Rueden, C., Saalfeld, S., Schmid, B., Tinevez, J.-Y., White, D. J., Hartenstein, V., Eliceiri, K., Tomancak, P., & Cardona, A. (2012). Fiji: an open-source platform for biological-image analysis. Nature Methods, 9(7), 676–682.

Journal of Experimental Biology • Supplementary information
